# BPC-157 as an Investigational Peptide Therapeutic: Biopharmaceutical Challenges, Formulation Strategies, and Translational Development Barriers

**DOI:** 10.3390/pharmaceutics18050625

**Published:** 2026-05-20

**Authors:** Diana-Maria Mateescu, Dragos-Mihai Gavrilescu, Florin Eugen Constantinescu, Cristian Oancea, Adrian-Cosmin Ilie, Roxana Folescu, Mihaela-Diana Popa, Stela Iurciuc, Camelia-Oana Muresan, Alexandra Enache

**Affiliations:** 1 Department of General Medicine, Doctoral School, “Victor Babes” University of Medicine and Pharmacy Timisoara, Eftimie Murgu Square 2, 300041 Timisoara, Romania; diana.mateescu@umft.ro; 2 Department of Orthodontics, Dental District, Zăgazului 3, One Floreasca Vista, Sector 1, 014261 Bucharest, Romania; dr.gavrilescu@outlook.com; 3 Department of Prosthodontics, Faculty of Dental Medicine, Carol Davila University of Medicine and Pharmacy, Eroii Sanitari Blvd 8, 050474 Bucharest, Romania; dr.florin.constantinescu@gmail.com; 4 Pulmonology Department, “Victor Babes” University of Medicine and Pharmacy Timisoara, 300041 Timisoara, Romania; oancea@umft.ro; 5 Department of Public Health and Sanitary Management, “Victor Babes” University of Medicine and Pharmacy Timisoara, Eftimie Murgu Square 2, 300041 Timisoara, Romania; ilie.adrian@umft.ro; 6 Centre for Translational Research and Systems Medicine, Faculty of Medicine, “Victor Babes” University of Medicine and Pharmacy Timisoara, Eftimie Murgu Sq. No. 2, 300041 Timisoara, Romania; 7 Department of Balneology, Medical Recovery and Rheumatology, Family Discipline, Center for Preventive Medicine, “Victor Babes” University of Medicine and Pharmacy Timisoara, Eftimie Murgu Square 2, 300041 Timisoara, Romania; folescu.roxana@umft.ro; 8 Department of Microbiology, “Victor Babes” University of Medicine and Pharmacy Timisoara, Eftimie Murgu Square 2, 300041 Timisoara, Romania; popa.mihaela@umft.ro; 9 Cardiology Department, “Victor Babes” University of Medicine and Pharmacy Timisoara, Eftimie Murgu Square 2, 300041 Timisoara, Romania; 10 Legal Medicine, Timisoara Institute of Legal Medicine, 300041 Timisoara, Romania; enache.alexandra@umft.ro; 11 Ethics and Human Identification Research Center, “Victor Babes” University of Medicine and Pharmacy Timisoara, Eftimie Murgu Square 2, 300041 Timisoara, Romania; 12 Discipline of Forensic Medicine, Bioethics, Deontology, and Medical Law, Department of Neuroscience, “Victor Babes” University of Medicine and Pharmacy Timisoara, Eftimie Murgu Square 2, 300041 Timisoara, Romania

**Keywords:** BPC-157, peptide therapeutics, biopharmaceutical classification system, formulation strategies, pharmacokinetic–pharmacodynamic disconnect, translational barriers, drug development, oral peptide delivery, compounding regulation, narrative review

## Abstract

**Background/Objectives**: BPC-157 (body protection compound 157) is a synthetic pentadecapeptide derived from a gastric protein fragment with reported cytoprotective and regenerative properties across multiple organ systems. Despite over three decades of preclinical research demonstrating consistent biological activity, its pharmaceutical development remains rudimentary, with no approved formulation, no validated dosing regimen, and no completed Phase II clinical trial. This review critically evaluates BPC-157 from a biopharmaceutical and drug development perspective, examining its physicochemical and pharmacokinetic properties, formulation challenges across routes of administration, the pharmacokinetic–pharmacodynamic disconnect that characterizes its preclinical profile, and the regulatory and translational barriers that currently preclude clinical advancement. **Methods**: A narrative review of the literature was conducted using PubMed/MEDLINE, Embase, and Cochrane Library from database inception to April 2026. Search terms included “BPC-157”, “BPC157”, “body protection compound 157”, “pentadecapeptide”, and “GEPPPGKPADDAGLV”, each combined with “pharmacokinetics”, “formulation”, “biopharmaceutics”, “drug delivery”, “clinical trial”, “toxicology”, and “regulatory”. Patent databases (Espacenet, Google Patents) and regulatory agency websites (FDA, EMA, WADA) were searched independently. Searches were supplemented by forward and backward citation tracking of key references. Articles were selected based on relevance to biopharmaceutical characterization, pharmacokinetics, formulation science, clinical evidence, and regulatory status; pharmacodynamic studies were included insofar as they inform translational development. Evidence was synthesized with emphasis on pharmaceutical characterization, formulation science, and translational feasibility; no formal quality assessment instrument was applied, consistent with the narrative review design. **Results**: BPC-157 exhibits unusual stability in gastric juice and demonstrates activity via oral, parenteral, and topical routes, yet its human pharmacokinetic profile remains critically undercharacterized despite a recently published formal preclinical ADME study in two species confirming a sub-30-min plasma half-life, linear dose-proportional kinetics, and intramuscular bioavailability of 14–51% depending on species. A plasma half-life of under 30 min—confirmed preclinically and in a preliminary two-subject human pilot—contrasts with prolonged biological effects lasting hours to days—a disconnect with significant implications for dosing strategy and formulation design. No pharmaceutical-grade formulation has been developed or validated. The peptide lacks bcs classification data, permeability characterization, and formal excipient compatibility studies. Available clinical data derive from fewer than 30 subjects across three uncontrolled pilot studies, none of which employed standardized pharmaceutical preparations. **Conclusions**: BPC-157 presents a compelling but pharmaceutically underdeveloped profile. The primary barrier to clinical translation is not the absence of biological activity, but the absence of fundamental pharmaceutical science: characterized formulations, validated pharmacokinetics, and a coherent drug development strategy. Addressing these biopharmaceutical gaps is a prerequisite for any meaningful clinical program.

## 1. Introduction

Body Protection Compound-157 (BPC-157) is a synthetic 15-amino acid peptide with the sequence GEPPPGKPADDAGLV and a molecular weight of approximately 1419 Da. The designation “BPC” refers to “Body Protection Compound,” a series of peptide fractions isolated and screened by Sikiric and colleagues at the University of Zagreb in the early 1990s; the numeral “157” identifies this specific sequence as the 157th candidate compound from that original screening programme [1,2]. BPC-157 was derived from a naturally occurring protein fragment present in human gastric juice: fractionation of gastric secretions yielded a family of cytoprotective peptides, and BPC-157 represents the synthetic counterpart of one such sequence, subsequently confirmed to be stable under the acidic and proteolytic conditions of the gastric environment [1,2]. Since its initial characterization, BPC-157 has been investigated in an expanding body of predominantly preclinical studies spanning gastrointestinal, musculoskeletal, cardiovascular, neurological and ophthalmic domains [1,2,3,4,5]. Across diverse experimental models, it has been consistently associated with cytoprotective, angiogenic, and regenerative effects, particularly in the context of tissue injury and inflammation [2,3,6].

From a pharmaceutical perspective, BPC-157 exhibits several properties that distinguish it from conventional peptide drug candidates. Notably, it demonstrates resistance to enzymatic and acidic degradation under gastric conditions, a characteristic uncommon among peptide therapeutics, which are typically rapidly degraded in the gastrointestinal tract [1,6,7,8]. This observation has led to the hypothesis that oral administration may be feasible, although this remains unverified in rigorously designed pharmacokinetic studies. In addition, biological activity has been reported following parenteral and topical administration, suggesting potential flexibility in delivery routes [2,9]. Preclinical toxicological evaluations have not identified a defined lethal dose, and no consistent organ-level toxicity signals have been reported, although these findings remain limited to non-clinical settings [2,10].

Despite these favorable characteristics, BPC-157 has not progressed beyond the earliest stages of pharmaceutical development. No pharmaceutical-grade formulation has been standardized or validated, and no comprehensive human pharmacokinetic studies have been conducted to characterize its absorption, distribution, metabolism, and elimination. Current dosing regimens used in experimental settings—and, in some cases, extrapolated to off-label human use—are derived primarily from animal studies, without formal allometric scaling or pharmacokinetic validation [2,10]. A Phase I clinical trial (NCT02637284), which could have provided foundational pharmacokinetic and safety data, was terminated without publication of results, leaving a substantial and persistent evidence gap [10].

The regulatory implications of this developmental immaturity are significant. BPC-157 has been subject to regulatory scrutiny by the U.S. Food and Drug Administration, reflecting insufficient human safety and efficacy data for compounding use [11]. Similarly, it has been referenced within the World Anti-Doping Agency framework as an unapproved substance in the context of performance enhancement [12]. Despite these signals, interest in BPC-157 has continued to grow, accompanied by increasing non-regulated use and informal distribution through online and gray-market channels [2,10].

This disconnect between extensive preclinical research, regulatory uncertainty, and ongoing non-regulated use defines the central problem addressed in this review. While the pharmacodynamic effects of BPC-157 across organ systems have been extensively described in the literature [2,7], its biopharmaceutical characterization remains critically underdeveloped. Accordingly, this review focuses on the physicochemical, pharmacokinetic, and translational barriers that currently limit its development as a legitimate pharmaceutical agent.

## 2. BPC-157: Physicochemical Profile and Structural Basis of Stability

BPC-157 (GEPPPGKPADDAGLV) is a linear synthetic pentadecapeptide with a molecular weight of approximately 1419 Da, placing it within the lower range of therapeutic peptide candidates currently under pharmaceutical development [7,13]. Its amino acid composition is characterized by an unusually high proline content—three consecutive proline residues at positions 3–5—which confers significant conformational rigidity to the N-terminal segment of the molecule [1,14]. Proline, as a cyclic imino acid, restricts backbone dihedral angles and limits the conformational flexibility that renders most linear peptides susceptible to proteolytic recognition and cleavage [6,8]. This structural feature is considered a primary determinant of BPC-157’s reported resistance to enzymatic degradation under gastric conditions, distinguishing it from the majority of peptide drug candidates, which require chemical modification or formulation protection to survive the gastrointestinal environment [3,15].

The peptide carries a net neutral to slightly positive charge at physiological pH, attributable to the lysine residue at position 7 (pKa ~10.5) and the absence of strongly acidic residues in the C-terminal domain [14,16]. This charge profile has implications for both membrane permeability and formulation behavior: the relatively low net charge reduces electrostatic repulsion at mucosal surfaces, potentially facilitating passive transcellular diffusion, while the amphipathic character of the sequence—hydrophilic N-terminal and more hydrophobic C-terminal residues—may support interaction with lipid bilayers at physiologically relevant concentrations [6,17]. However, these properties have not been formally characterized through standard permeability assays such as Caco-2 or PAMPA models, representing a fundamental gap in the biopharmaceutical dataset for this compound.

Aqueous solubility of BPC-157 has been reported to be adequate for parenteral formulation at therapeutic dose ranges, though no formal solubility profiling across physiologically relevant pH values (1.2, 4.5, 6.8, 7.4) has been published [14,17]. Such profiling is a standard requirement under ICH Q6B guidelines for peptide drug substances and would be necessary to support any regulatory submission. Similarly, no data exist on the peptide’s stability in simulated intestinal fluid, its partition coefficient (logP or logD), or its binding to plasma proteins—parameters that collectively determine the biopharmaceutical feasibility of a given route of administration and that are routinely characterized early in peptide drug development [17,18].

The reported stability of BPC-157 in human gastric juice is its most pharmacologically distinctive physicochemical property and the one most frequently cited to justify oral administration [1,2]. Native gastric juice at pH 1–2 contains pepsin at concentrations of 0.5–1 mg/mL, along with hydrochloric acid and various proteases, conditions under which the vast majority of therapeutic peptides are rapidly hydrolyzed [3,6]. BPC-157’s resistance to this environment has been demonstrated empirically in in vitro incubation studies, with the intact peptide recoverable after prolonged exposure periods [1]. The mechanistic basis of this stability is most plausibly attributed to the conformational constraints imposed by the N-terminal polyproline segment, which may adopt a polyproline II helix—a left-handed helical structure known to be resistant to proteolytic enzymes due to steric occlusion of the peptide backbone [8]. However, this structural hypothesis has not been confirmed by circular dichroism, nuclear magnetic resonance spectroscopy, or X-ray crystallography, and the three-dimensional solution structure of BPC-157 remains uncharacterized in the peer-reviewed literature [14,16].

It should be noted that gastric stability, while necessary, is not sufficient to establish oral bioavailability for a peptide therapeutic [3,15,19]. Intestinal stability, epithelial permeability, first-pass hepatic metabolism, and lymphatic absorption represent additional sequential barriers, each of which must be characterized and addressed in a comprehensive biopharmaceutical development program [17]. The oral peptide therapeutics that have successfully reached clinical approval—including semaglutide (Rybelsus^®^), cyclosporine, and desmopressin—achieved this through combinations of chemical modification, permeation enhancement, or specialized formulation strategies that go substantially beyond reliance on intrinsic stability alone [3,20]. Whether BPC-157’s intrinsic properties are sufficient to support clinically meaningful oral bioavailability, or whether formulation intervention will be required, cannot be determined from the available data.

Table 1 below summarises the current stability knowledge base for BPC-157 across relevant compartments and degradation pathways. Gastric stability is the compound’s most distinctive and well-documented property: BPC-157 survives exposure to gastric juice at pH 1–2 with pepsin, in contrast to the vast majority of therapeutic peptides [1,2]. Plasma stability is poor, with a confirmed half-life of less than 30 min in both preclinical species and the only available human study, driven by rapid renal filtration and proteolytic hydrolysis [10,21]. Intestinal stability and hepatic first-pass extraction remain entirely uncharacterised. From a chemical degradation standpoint, the sequence is unusually favourable: the absence of methionine, cysteine, tryptophan, asparagine, and glutamine eliminates the principal oxidative and deamidation pathways that dominate peptide formulation development, leaving aspartyl-bond hydrolysis at the Asp10–Asp11 junction as the primary chemical liability under acidic and neutral conditions [14,17]. Physical stability (aggregation, adsorption) is uncharacterised. This profile—high gastric stability combined with poor systemic half-life and acceptable chemical degradation risk—has specific and actionable implications for formulation strategy: oral delivery targeting the gastric window is mechanistically justified, and parenteral formulations do not face the oxidative or deamidation hurdles common to many peptide drug substances.

**Figure 1:**
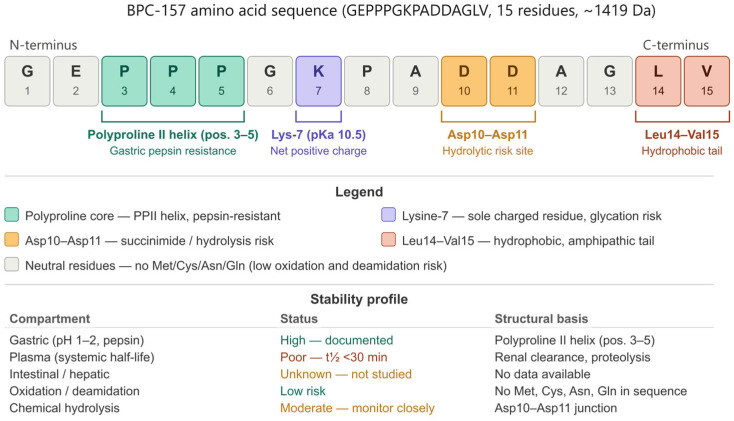
Amino acid sequence map of BPC-157 (GEPPPGKPADDAGLV). Residues are color-coded by functional role: the N-terminal polyproline cluster (positions 3–5, green) confers resistance to pepsin and gastric proteolysis via a polyproline II helix; lysine at position 7 (purple) is the sole charged residue at physiological pH (pKa ~10.5) and introduces a glycation liability in the presence of reducing-sugar excipients; the Asp10–Asp11 junction (amber) represents the principal hydrolytic and succinimide formation risk under acidic and neutral conditions; the C-terminal leucine–valine dipeptide (positions 14–15, coral) contributes hydrophobic and amphipathic character. Neutral residues (gray) contain no methionine, cysteine, asparagine, or glutamine, resulting in a favorable oxidative and deamidation stability profile. The lower panel summarizes the stability profile across pharmacologically relevant compartments. Da: daltons; t½: elimination half-life.

**Figure 2:**
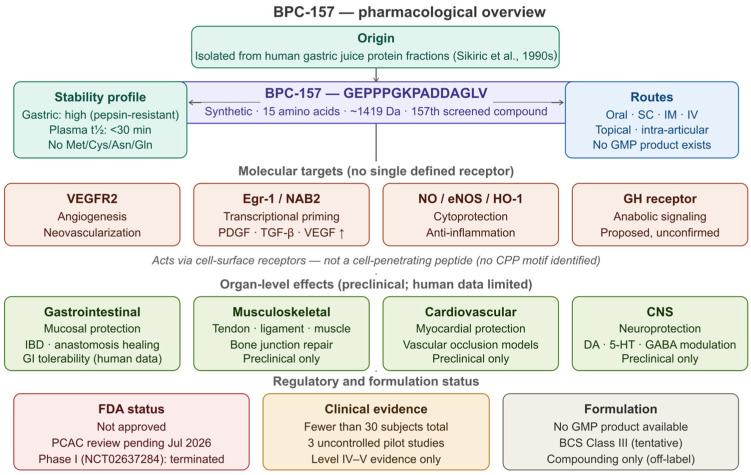
Pharmacological overview of BPC-157. The diagram illustrates the compound’s origin from human gastric juice protein fractions [2], its core structural identity (GEPPPGKPADDAGLV, 15 amino acids, ~1419 Da), and key biopharmaceutical properties including gastric stability and available administration routes. The central tier shows the four principal molecular signaling targets: vascular endothelial growth factor receptor 2 (VEGFR2), early growth response gene 1 (Egr-1) and its co-regulator NAB2, the nitric oxide system (NO/eNOS/HO-1), and the growth hormone receptor (proposed). BPC-157 acts via cell-surface receptors and is not a cell-penetrating peptide. Organ-level effects documented in preclinical models are shown for gastrointestinal, musculoskeletal, cardiovascular, and central nervous system domains. The lower tier summarizes the current regulatory and formulation status. 5-HT: serotonin; BCS: Biopharmaceutical Classification System; CNS: central nervous system; CPP: cell-penetrating peptide; DA: dopamine; eNOS: endothelial nitric oxide synthase; GH: growth hormone; GMP: Good Manufacturing Practice; HO-1: heme oxygenase-1; IBD: inflammatory bowel disease; IM: intramuscular; IV: intravenous; NAB2: NGFI-A binding protein 2; NO: nitric oxide; PCAC: Pharmacy Compounding Advisory Committee; SC: subcutaneous; VEGFR2: vascular endothelial growth factor receptor 2. Arrows indicate the conceptual and directional relationships between the origin, structural properties, molecular targets, organ-level effects, and regulatory status of BPC-157.

In summary, BPC-157 possesses a physicochemical profile that is pharmacologically interesting but biopharmaceutically incomplete. Its polyproline-driven conformational stability represents a genuine and unusual asset among peptide drug candidates. However, the absence of formal characterization across standard biopharmaceutical parameters—solubility profiling, permeability assays, plasma protein binding, intestinal stability, and solution structure determination—means that its development potential cannot be rigorously assessed. These characterization gaps are not incidental; they reflect the broader absence of a structured pharmaceutical development program for this compound and constitute the primary biopharmaceutical barrier to its advancement [9,13,16]. The amino acid sequence of BPC-157, key functional regions, and their pharmaceutical implications are illustrated in Figure 1. The sequence map and functional region annotations in Figure 2 provide a visual reference for these stability-relevant structural features.

## 3. Pharmacological Background: Mechanisms and Organ-Specific Effects

Given that the pharmacodynamic profile of BPC-157 has been extensively reviewed elsewhere [1,2,3,6,7], the present section provides a condensed synthesis of its principal mechanisms of action and organ-specific effects, intended to contextualize the biopharmaceutical and translational analysis that follows. Readers seeking comprehensive pharmacological detail are referred to recent systematic and narrative reviews [1,2,3,6,7]. A schematic overview of BPC-157’s origin, molecular targets, organ-level effects, and current regulatory and formulation status is presented in Figure 2.

### 3.1. Molecular Mechanisms of Action

BPC-157 exerts its biological effects through multiple, partially overlapping signaling pathways rather than through a single defined receptor mechanism—a feature that distinguishes it from conventional small-molecule therapeutics and complicates its pharmacological classification [1,2]. The most consistently documented molecular targets include vascular endothelial growth factor receptor 2 (VEGFR2), the early growth response gene 1 (Egr-1) transcription factor, the nitric oxide (NO) system, and growth hormone (GH) receptor signaling [1,2].

VEGFR2 activation by BPC-157 promotes endothelial cell proliferation and migration, resulting in neovascularization at sites of tissue injury [22,23]. This angiogenic response is mechanistically supported by upregulation of VEGF expression and activation of the FAK-paxillin signaling axis, which facilitates organized cell migration and extracellular matrix remodeling at wound sites [24,25]. Egr-1, a zinc-finger transcription factor functioning as a master regulator of tissue repair gene networks, is also upregulated by BPC-157, driving downstream expression of PDGF, TGF-β, and VEGF and amplifying the regenerative cascade [26,27]. The interaction with the NO system is complex and context-dependent: BPC-157 positively modulates endothelial NOS (eNOS) activity and upregulates heme oxygenase-1 (HO-1), an antioxidant enzyme with cytoprotective and anti-inflammatory properties [28,29]. Strategies to therapeutically increase nitric oxide signalling have been extensively explored in cardiovascular disease [30,31]. Notably, BPC-157’s cytoprotective effects have been shown to persist under conditions of pharmacological NOS blockade, suggesting mechanistic redundancy beyond NO-dependent pathways [1,2]. Anti-inflammatory activity is mediated in part through suppression of pro-inflammatory cytokines including TNF-α, IL-1β, and IL-6, with downstream modulation of NF-κB signaling implicated as a probable contributing mechanism [32,33].

No high-affinity receptor binding site for BPC-157 has been identified, and its molecular target remains formally unknown [1,2]. This absence of a defined receptor is a significant limitation not only for mechanistic understanding but for pharmaceutical development: without a characterized binding site, receptor occupancy-based PK/PD modeling—the standard framework for dose–response characterization in drug development—cannot be applied [18]. Growth hormone receptor upregulation has been proposed as an additional mechanism, potentially amplifying endogenous anabolic signaling in musculoskeletal tissues without directly increasing circulating GH levels [2], though this interaction has not been characterized at the molecular level.

A question of particular relevance to both mechanistic understanding and formulation design is whether BPC-157 requires cell penetration to exert its biological effects. BPC-157 does not bear the structural hallmarks of cell-penetrating peptides (CPPs): it lacks the poly-cationic or amphipathic motifs (such as the Arg-rich sequences of Tat or penetratin) that define established CPP classes, and its sequence analysis does not predict membrane-translocating activity [1,2]. The primary documented molecular targets of BPC-157—VEGFR2 and the growth hormone receptor—are transmembrane receptor tyrosine kinases and G-protein-coupled receptors that are activated at the cell surface; their downstream signaling cascades, including FAK-paxillin, NO-system modulation, and Egr-1 transcriptional activation, are initiated extracellularly and do not require cytosolic delivery of the peptide [1,2,22,24]. The nuclear transcription factor Egr-1, though its ultimate action is intranuclear, is activated downstream of surface receptor signaling and does not require direct BPC-157 entry into the cell. Accordingly, standard peptide delivery approaches—aqueous parenteral formulations, oral delivery with absorption enhancement, and local topical or intra-articular administration—are expected to be sufficient for biological activity, without the need for specialized cell-penetration technologies such as endosomal escape agents, fusogenic lipids, or CPP conjugation. This conclusion is consistent with the documented biological activity of BPC-157 following conventional intraperitoneal, intragastric, and subcutaneous administration in preclinical models [1,2,7,21], where no cell-penetration enhancement was employed. The absence of CPP character simplifies formulation strategy considerably relative to peptide therapeutics that must achieve intracellular delivery.

### 3.2. Organ-Specific Effects: A Summary

Musculoskeletal system. BPC-157 has been investigated in a larger body of preclinical musculoskeletal research than any other organ system [7]. In rodent models of Achilles tendon transection, ligament injury, skeletal muscle laceration, and fracture, it consistently demonstrated accelerated structural repair, improved biomechanical parameters, and enhanced functional recovery [1,7]. The primary mechanisms in connective tissue involve Egr-1-driven fibroblast activation, VEGF-mediated neovascularization of avascular tissue compartments, and TGF-β-mediated collagen type I synthesis [26,34]. A 2025 systematic review identified 36 relevant studies, of which 35 were preclinical and one was a small retrospective clinical analysis, confirming consistent preclinical efficacy but highlighting the near-complete absence of human data [7].

Gastrointestinal system. As the organ system from which BPC-157 was originally derived, the gastrointestinal tract represents its most extensively characterized therapeutic domain [1,2]. Cytoprotective effects have been demonstrated across the entire length of the gastrointestinal tract, encompassing alcohol- and NSAID-induced gastric mucosal injury, inflammatory bowel disease models, intestinal anastomosis healing, esophageal mucosal damage, and lower esophageal sphincter dysfunction [1,2,35,36,37]. Early-phase clinical investigations were conducted under the designations PL-10, PLD-116, and PL14736 by Pliva (Croatia) for inflammatory bowel disease, providing limited but preliminary human tolerability data [1,2]. These represent the only clinical investigations of BPC-157 in a gastrointestinal indication to date and have not been followed by Phase II or III trials.

Central nervous system. Peripheral administration of BPC-157 consistently produces central neurochemical changes across dopaminergic, serotonergic, and GABAergic systems in rodent models, a pattern that implies either central penetration or peripheral-to-central signaling mechanisms that have not been formally characterized [2,38]. Neuroprotective effects have been demonstrated in models of global cerebral ischemia, spinal cord compression, and traumatic brain injury, with histological evidence of reduced axonal necrosis, attenuated demyelination, and preserved neuronal architecture [2]. The 2024 review by Vukojević and colleagues proposed a cytoprotective neurotransmitter framework to account for BPC-157’s bidirectional modulatory effects across neurotransmitter systems, positing a homeostatic rather than agonist or antagonist mechanism of CNS action [2]. No human CNS data exist for BPC-157 in any neurological indication.

### 3.3. Methodological Limitations of the Preclinical Evidence Base

Any biopharmaceutical evaluation of BPC-157 must account for the methodological characteristics of its underlying evidence base, as these directly bear on the validity of translational inferences. Several limitations warrant explicit acknowledgment.

First, the overwhelming majority of published BPC-157 studies—exceeding 80% by author affiliation analysis—originate from a single research group at the University of Zagreb, led by Sikiric and colleagues [1,2]. While the internal consistency of this body of work is notable, the absence of independent replication across geographically and institutionally diverse laboratories represents a significant limitation by contemporary standards of preclinical evidence evaluation [1,7]. Independent replication is considered essential before preclinical findings can be considered sufficiently robust to justify human clinical investigation.

Second, virtually all preclinical studies employ a single dose level—typically 10 µg/kg or 10 ng/kg administered intraperitoneally or intragastrically—precluding the construction of dose–response relationships and the identification of minimum effective concentrations, maximum tolerated doses, or therapeutic windows [1,10]. This is a critical gap for pharmaceutical development, where dose–response characterization is a regulatory prerequisite for clinical dose selection.

Third, the predominant use of rodent models, while standard in early-stage pharmacological research, introduces well-recognized limitations in translational predictivity, particularly for peptide therapeutics where species differences in proteolytic enzyme activity, gastrointestinal physiology, and renal clearance can substantially alter pharmacokinetic and pharmacodynamic behavior [17,18]. Allometric scaling of BPC-157 dosing from rodent to human has not been formally performed or published.

Fourth, no study to date has characterized the relationship between plasma BPC-157 concentrations and observed biological effects—a fundamental requirement for PK/PD modeling and rational dose selection in clinical development [18]. While He and colleagues [21] employed an LC-MS/MS method for BPC-157 quantification in rat and dog plasma, this method has not been validated against FDA Bioanalytical Method Validation guidance for human matrices, and no published study has characterized BPC-157 concentrations in human plasma following therapeutic dosing with full analytical transparency—representing a prerequisite gap that must be addressed before pharmacokinetic data acceptable to regulatory agencies can be generated.

These limitations do not invalidate the preclinical evidence base, but they define its boundaries and establish the minimum scientific work required before clinical translation can be responsibly pursued.

## 4. Pharmacokinetic Profile and the Pharmacokinetic–Pharmacodynamic Disconnect

### 4.1. Overview of Peptide Pharmacokinetics: A Developmental Framework

The pharmacokinetic characterization of therapeutic peptides presents challenges that differ fundamentally from those encountered with small-molecule drugs, and an understanding of these challenges is necessary to contextualize the pharmacokinetic data available for BPC-157 [9,13]. Peptides are subject to proteolytic degradation at multiple sites—the gastrointestinal lumen, the intestinal epithelium, the systemic circulation, and target tissues—resulting in rapid elimination half-lives that rarely exceed 30–60 min for unmodified linear sequences [17,18]. Renal filtration represents an additional major elimination pathway for peptides below approximately 30 kDa, contributing to rapid systemic clearance [17]. The pharmacokinetic consequences of these elimination mechanisms—short half-lives, low oral bioavailability, and high clearance rates—have historically driven the pharmaceutical industry toward chemical modification strategies including PEGylation, lipidation, cyclization, and amino acid substitution to produce developable drug candidates with adequate exposure profiles [39,40].

Against this backdrop, BPC-157 presents a pharmacokinetic profile that is simultaneously intriguing and poorly characterized. Its reported gastric stability suggests potential oral bioavailability, but its small size, linear structure, and absence of half-life extension modifications predict rapid systemic elimination—a combination that creates fundamental tensions for dosing strategy and formulation design [1,3,9]. The resolution of these tensions requires pharmacokinetic data of a quality and quantity that, until recently, did not exist for this compound. The publication of a formal preclinical ADME study by He and colleagues [21] represents an important first step, although the absence of human oral and subcutaneous pharmacokinetic data remains a critical gap for clinical translation.

### 4.2. Available Pharmacokinetic Data

The first formal pharmacokinetic characterization of BPC-157 was reported by He and colleagues in a comprehensive ADME study conducted in Sprague-Dawley rats and beagle dogs (*n* = 6 per group per timepoint), representing the only published study to characterize the full pharmacokinetic profile of BPC-157 in any species under regulatory-grade study conditions [21]. Following single intravenous administration, the elimination half-life (t½) of BPC-157 was 15.2 min in rats and 5.27 min in beagle dogs, consistent with rapid renal and proteolytic clearance characteristic of unmodified linear peptides [21]. Following intramuscular administration across three escalating dose levels—20, 100, and 500 µg/kg in rats and 6, 30, and 150 µg/kg in dogs—BPC-157 demonstrated linear pharmacokinetic behavior, with proportional increases in Cmax and AUC across the full dose range and no significant accumulation on seven-day repeat dosing [21]. Absolute intramuscular bioavailability was 14–19% in rats and 45–51% in beagle dogs—a substantial inter-species difference with direct implications for allometric dose scaling, as discussed in Section 4.4. Tissue distribution studies using [^3^H]-labeled BPC-157 demonstrated peak radioactivity concentrations in most tissues at approximately one-hour post-administration, with highest concentrations in the intestinal tract, followed by lung, gonad, skin, and skeletal muscle; brain and adipose tissue exhibited the lowest concentrations, suggesting limited central nervous system penetration under basal pharmacokinetic conditions [21]. Metabolic fate was characterized as rapid proteolytic hydrolysis to six identified small peptide fragments (M1–M6), subsequently converted to free amino acids entering endogenous metabolic pathways; primary excretion routes were urinary and biliary [21]. The bioanalytical method employed was liquid chromatography coupled with tandem mass spectrometry (LC-MS/MS), providing the quantitative sensitivity and specificity appropriate for a regulatory-grade pharmacokinetic investigation [21]. Importantly, He and colleagues represent an independent research group—affiliated with the Air Force Medical University and Shaanxi University of Chinese Medicine, Xi’an, China—providing the first pharmacokinetic dataset for BPC-157 generated outside the University of Zagreb research programme.

The only available human pharmacokinetic data derive from a two-subject pilot study by Lee and Burgess [10], in which a 58-year-old male and a 68-year-old female received intravenous infusions of 10 mg and 20 mg BPC-157 on consecutive days. Serial plasma sampling demonstrated a half-life of under 30 min, with plasma concentrations returning to baseline within 24 h and primary renal clearance—findings directionally concordant with the preclinical ADME dataset of He and colleagues [21]. No adverse events were observed across either dose level [10]. While this preliminary concordance between human and preclinical half-life estimates is pharmacokinetically consistent with renally-mediated peptide elimination and suggests that the rat and beagle dog models may offer reasonable translational predictivity for systemic clearance, the limitations of this dataset are substantial and must be stated explicitly. A sample size of two subjects is statistically insufficient to characterize inter-individual pharmacokinetic variability; the enrolled age range (58–68 years) introduces age-related renal function decline as a confounding variable; intravenous administration does not characterize the absorption phase critical for oral or subcutaneous formulation development; and the bioanalytical methodology was not fully reported, precluding independent assessment of data quality [10]. The study was published in a journal with a limited impact factor, further constraining the weight it can carry in regulatory and scientific contexts. Volume of distribution, plasma protein binding, oral bioavailability, subcutaneous absorption kinetics, and metabolite identification in humans all remain uncharacterized. Taken together, Lee and Burgess [10] provide preliminary directional confirmation of the preclinical PK profile but is insufficient to support clinical dose selection, formulation development, or regulatory submission for any indication.

The combined preclinical and preliminary human dataset establishes several pharmacokinetic parameters with reasonable confidence—sub-30-min systemic half-life, linear dose-exposure relationship, renal and biliary excretion, and rapid proteolytic metabolism to amino acid constituents—while leaving critical gaps that must be resolved before clinical translation can be pursued. Specifically absent from the current pharmacokinetic database are: oral bioavailability in any species under standardized fasting and fed conditions; subcutaneous pharmacokinetic characterization; volume of distribution and plasma protein binding data; a validated bioanalytical method accepted under FDA and EMA guidance; and any population pharmacokinetic data incorporating clinically relevant covariates such as renal function, body composition, and age [17,18,21]. The inter-species bioavailability difference—14–19% IM in rats versus 45–51% in dogs—highlights the uncertainty inherent in human dose prediction from preclinical data alone and reinforces the necessity of formal human pharmacokinetic characterization as a prerequisite for any clinical development programme.

In the preclinical setting, the pharmacokinetic dataset for BPC-157 remains limited to the formal ADME characterization by He and colleagues [21], which employed an LC-MS/MS bioanalytical method and represented a significant methodological advance over earlier pharmacological studies. However, this method has not been independently validated against FDA Bioanalytical Method Validation guidance and EMA equivalents for use in human matrices, and the study was not conducted under full Good Laboratory Practice (GLP) conditions as required for IND submission [11,17]. Without a fully validated, regulatory-grade bioanalytical method, concentration-time profiles sufficient for regulatory submission cannot be generated, and this gap constitutes a prerequisite requirement before pharmacokinetic data acceptable to regulatory agencies can be produced [11,17].

### 4.3. The Pharmacokinetic–Pharmacodynamic Disconnect

The most pharmaceutically consequential feature of BPC-157’s profile is the dissociation between its sub-30-min plasma half-life and the persistence of biological effects measured days to weeks after a single dose [1,2,18,21]. The four candidate mechanisms commonly invoked to account for such PK/PD disconnects in peptide therapeutics—slow receptor dissociation, transcriptional priming, preferential tissue accumulation, and downstream biology slower than the pharmacokinetic exposure window—are not equally supported by the BPC-157 data, and we evaluate them here in order of decreasing consistency with the published evidence.

Most consistent with available data: indirect pharmacodynamic mechanisms. The biological endpoints most frequently used to characterise BPC-157 activity—neovascularisation, collagen deposition, axonal regrowth, anastomotic strength, and tissue remodelling [22,24,25,34,41]—are processes whose kinetics are governed by the cell biology of repair rather than by the residence time of the initiating signal. Endothelial proliferation and capillary sprouting unfold over 3–7 days; collagen type-I matrix maturation extends over 1–3 weeks; axonal regeneration spans weeks. A transient pharmacokinetic exposure can plausibly trigger such cascades and then become irrelevant to their progression. This framework is consistent with the observation that single-dose BPC-157 administration in injury models produces sustained histological recovery without the need for prolonged plasma exposure [1,2,7], and it is the most parsimonious explanation in that it requires no unverified molecular assumption.

Strongly consistent: transcriptional priming via Egr-1 and downstream growth-factor networks. BPC-157 has been documented to upregulate Egr-1 and its NAB2 regulatory partner, with downstream transcriptional consequences including upregulation of growth-factor genes implicated in wound healing [26,27]. Once initiated, transcriptional programmes of this class are self-sustaining through autocrine and paracrine signalling and do not require continued ligand presence. This mechanism is partially supported by direct gene-expression data—distinguishing it from receptor-binding hypotheses for which no molecular evidence exists—and overlaps mechanistically with the indirect-biology framework above. Whether the transcriptional signature observed in vitro persists in vivo for the duration required to drive the long pharmacodynamic tail has not been formally tested and represents a tractable experimental question.

Plausible but unverified: preferential tissue distribution and local accumulation. The [^3^H]-BPC-157 tissue-distribution data reported by He and colleagues [21] demonstrate peak tissue radioactivity at approximately one-hour post-administration, with the highest signal in intestinal tract, lung, gonad, skin, and skeletal muscle, and the lowest in brain and adipose tissue. These data establish that BPC-157 distributes broadly into peripheral tissues but do not demonstrate prolonged tissue retention beyond the systemic exposure window—terminal tissue concentrations were not characterised, and no compartmental analysis was performed [21]. Whether vascular permeability at sites of inflammation produces local concentrations that meaningfully exceed and outlast systemic clearance therefore remains an open question. This hypothesis is currently neither supported nor refuted by the published data and would require [^3^H]-BPC-157 administration in injury models with extended post-dose tissue sampling.

Least consistent: slow receptor dissociation kinetics. The classical PK/PD-disconnect mechanism for small-molecule and biologic drugs—sustained signalling driven by long receptor occupancy—requires identification of the receptor and quantification of its binding kinetics. No high-affinity receptor for BPC-157 has been identified after three decades of investigation [1,2]. The proposed interaction with the growth hormone receptor remains uncharacterised at the molecular level [2], and the absence of a binding-site model precludes any meaningful evaluation of dissociation kinetics. This hypothesis is therefore not currently testable and is the least supported of the four. It cannot be excluded, but it cannot inform formulation strategy or dosing design until the molecular target is defined.

Implications for formulation strategy. Ranking these mechanisms matters because they imply different formulation priorities. If the indirect-biology and transcriptional-priming hypotheses are correct—as the available data most strongly suggest—then intermittent dosing with conventional immediate-release formulations is likely to be adequate, and the rationale for modified-release parenteral systems weakens substantially. If preferential tissue accumulation contributes meaningfully, local-delivery strategies (intra-articular, intra-tendinous, topical) gain priority over systemic modified-release formulations. If receptor-occupancy kinetics dominate—currently unverifiable—formulation choice would depend on binding-site characterisation that does not yet exist. Resolving which mechanism predominates is therefore not an academic question; it is a prerequisite for rational formulation strategy, and the experiments required (extended tissue PK, in vivo Egr-1 time-course, target deconvolution) are well within the reach of standard pharmaceutical development methodology [18]. Taken together, the indirect pharmacodynamic mechanisms and Egr-1-driven transcriptional priming represent the explanations most consistent with the existing data. These do not require sustained plasma exposure and align with the observed prolonged tissue-level effects following single-dose administration. This interpretation substantially reduces the need for complex modified-release formulations and supports intermittent dosing regimens in future clinical studies.

### 4.4. Allometric Scaling and the Absence of Human Dose Justification

The dosing regimens currently employed in off-label human use of BPC-157—typically in the range of 200–500 µg per day administered subcutaneously or orally—are derived by informal extrapolation from the rodent dose range of 10 µg/kg to 10 ng/kg, without application of formal allometric scaling principles [1,10]; notably, He and colleagues proposed a clinical dose of 200 µg/person/day based on body surface area conversion from the rat effective dose range of 6–50 µg/kg [21], representing the only published attempt at formal dose translation, though this conversion requires validation against human pharmacokinetic data. Allometric scaling for peptide therapeutics is substantially more complex than for small molecules, because peptide clearance is driven by multiple mechanisms—renal filtration, proteolytic degradation, receptor-mediated endocytosis—whose allometric exponents differ and must be considered independently [17,18]. Simple body weight scaling systematically underestimates clearance differences between species for renally eliminated compounds, and the resulting human dose estimates can deviate from pharmacokinetically justified values by an order of magnitude or more [18].

The consequence of this absence of formal dose justification is that current human exposures to BPC-157—whether through off-label clinical use or gray-market self-administration—occur at doses that are neither pharmacokinetically validated nor safety-anchored in human data [10,11]. This situation is not merely a scientific concern; it has direct regulatory implications, as dose selection for first-in-human studies requires explicit pharmacokinetic justification under current FDA and EMA guidance for peptide investigational products [11,17].

### 4.5. Implications for Clinical Development

The pharmacokinetic gaps identified above define a specific and actionable research agenda for BPC-157 clinical development. Priority requirements include: extension and regulatory-grade validation of the LC-MS/MS bioanalytical method reported by He and colleagues [21] for BPC-157 quantification in human plasma, urine, and relevant tissue matrices, with full compliance with FDA Bioanalytical Method Validation guidance and EMA equivalents; a formal single- and multiple-dose intravenous pharmacokinetic study in an adequately powered healthy volunteer population, including full ADME characterization; pharmacokinetic studies following oral and subcutaneous administration to characterize route-specific absorption and bioavailability; tissue distribution studies in relevant animal models; and formal allometric scaling to generate pharmacokinetically justified starting doses for first-in-human studies in patient populations [17,18]; allometric scaling validation using the inter-species bioavailability differential identified by He and colleagues (14–19% IM in rats versus 45–51% in dogs [21]) as a framework for predicting human subcutaneous exposure prior to first-in-human subcutaneous dosing studies. Additionally, population pharmacokinetic modeling incorporating covariates such as age, renal function, and body composition would be required to support dose individualization in clinical trials [18]. These requirements are not exceptional for a peptide drug candidate—they represent standard practice in contemporary peptide drug development [9,13,42]—but they highlight the substantial distance between BPC-157’s current state of pharmacokinetic characterization and the minimum data package required to initiate a credible clinical program.

## 5. Biopharmaceutical Classification and Route-of-Administration Analysis

### 5.1. Biopharmaceutical Classification of BPC-157: A Framework for Hypothesis Generation

No formal BCS classification data exist for BPC-157. The following assessment is presented strictly as a hypothesis-generating framework, intended to guide future experimental design rather than to establish classification status. All conclusions drawn are provisional and require validation through standardized regulatory-grade solubility and permeability assays as specified under FDA and EMA BCS guidance and the WHO Technical Report Series No. 1052 (2024). The Biopharmaceutical Classification System (BCS), originally developed by Amidon and colleagues and subsequently adopted by regulatory agencies worldwide as a framework for oral drug product development and biowaiver applications, categorizes drug substances according to two primary parameters: aqueous solubility across the physiological pH range and intestinal permeability [3,15,19]. For small molecules, BCS classification is a routine early-development activity that directly informs formulation strategy, predicts the rate-limiting step for oral absorption, and guides regulatory decision-making regarding the need for in vivo bioavailability studies [3]. For peptide therapeutics, BCS classification is more complex and less standardized, but the underlying framework remains conceptually applicable and analytically useful [15,17].

A formal BCS classification for BPC-157 has not been published. Based on available physicochemical and biological data, a tentative assessment can be proposed, subject to the substantial caveats imposed by the absence of rigorous experimental characterization. With respect to solubility, BPC-157 is a relatively small, charge-bearing peptide with a molecular weight of approximately 1419 Da and a net positive charge at physiological pH attributable to its lysine residue [1,14]. These properties are generally associated with adequate aqueous solubility across the gastrointestinal pH range, suggesting probable BCS high-solubility classification, though this has not been confirmed by formal dose-solubility ratio analysis as required under FDA and EMA BCS guidance [3,17]. With respect to permeability—the more critical and more uncertain parameter for peptide compounds—available evidence is insufficient to support a definitive classification [15,19].

Intestinal permeability for peptides is governed by a complex interplay of molecular size, charge, lipophilicity, hydrogen bonding capacity, and susceptibility to efflux transporters and intestinal metabolism [3,15]. For BPC-157, none of these determinants have been formally characterized through standard permeability assays. Its molecular weight of approximately 1419 Da substantially exceeds the empirical upper limit of approximately 500 Da associated with favorable passive transcellular permeability under Lipinski’s rule-of-five framework [6,16], though it is well established that this framework was derived from small-molecule datasets and has limited predictive validity for peptides [8]. Paracellular transport, which is size- and charge-restricted at intestinal tight junctions, is generally considered negligible for peptides above approximately 700 Da under physiological conditions [15,19]. Carrier-mediated transport via intestinal peptide transporters—primarily PepT1, which actively transports di- and tripeptides—is structurally incompatible with a 15-amino acid sequence [3]. Transcytosis via receptor-mediated or fluid-phase endocytosis pathways remains a theoretical possibility that has not been investigated for BPC-157 [15].

On the basis of these considerations, BPC-157 would most plausibly be classified as BCS Class III—high solubility, low permeability—by analogy with other small therapeutic peptides that demonstrate adequate solubility but limited intestinal absorption [3,19]. This tentative classification has direct implications for formulation strategy: BCS Class III compounds are absorption-limited rather than dissolution-limited, meaning that conventional formulation approaches focused on improving dissolution will not enhance oral bioavailability, and that permeability enhancement strategies—including the use of absorption enhancers, nanoparticulate carriers, or mucosal delivery systems—represent the appropriate development direction [3,15,19]. This classification must be considered provisional until formal solubility and permeability data are generated using standardized regulatory-grade assays.

### 5.2. Oral Administration: Opportunities, Barriers, and Comparators

The four sequential barriers to oral peptide bioavailability—gastric proteolysis, intestinal proteolysis, epithelial permeability, and hepatic first-pass metabolism—are addressed unevenly by BPC-157’s intrinsic properties. The peptide’s documented stability in human gastric juice [1,2] eliminates the first barrier, an asset shared by no other peptide drug candidate of comparable molecular weight and structural complexity. The remaining three barriers, however, are uncharacterised for BPC-157: no intestinal-stability data in simulated intestinal fluid or ex vivo intestinal preparations have been published [3,15]; no Caco-2 or PAMPA permeability assays have been performed [3,17]; and no hepatic extraction data exist [17,18]. The proposition that gastric stability alone is sufficient for clinically meaningful oral bioavailability—implicit in much of the BPC-157 oral-administration literature—is therefore unsupported by direct evidence and is contradicted by the precedent of approved oral peptides (oral semaglutide [3,20], cyclosporine [8,43], desmopressin), all of which required substantial pharmaceutical or chemical intervention beyond intrinsic stability.

The most consequential interpretive ambiguity in the BPC-157 oral-administration literature is the local-versus-systemic effect distinction. Biological effects observed following oral administration in rodent models—particularly those localised to the gastrointestinal tract—could reflect direct mucosal action of the intact peptide at the site of administration rather than systemic absorption [1,17]. This distinction has profound implications: a compound active locally in the gut without measurable systemic exposure is, pharmacologically, a topical mucosal therapeutic and must be developed as such, with formulation strategies (mucoadhesion, controlled-release matrices) and clinical positioning (IBD, NSAID enteropathy, mucosal repair) that differ fundamentally from those of a systemically absorbed oral peptide. The available BPC-157 literature does not consistently distinguish between these two scenarios, and the implications for indication selection have not been formally addressed.

Direct precedent for BPC-157 oral development is provided by oral semaglutide (Rybelsus^®^), which achieves systemic exposure via SNAC-mediated gastric absorption despite a comparable molecular weight [3,20], and by desmopressin, which achieves buccal and sublingual absorption at a molecular weight of 1069 Da [6]. Both precedents are instructive primarily by identifying the missing BPC-157-specific experiments: permeability of the gastric mucosa to BPC-157 in the presence of absorption enhancers has not been measured; the dose required to achieve therapeutic systemic exposure via a gastric-window route is unknown; and whether the polyproline-helix conformation that BPC-157 adopts in solution is preserved or disrupted under the local pH conditions created by SNAC co-formulation has not been assessed. These are the specific data gaps that must be resolved before the gastric-window strategy can be advanced beyond analogy.

Together, these considerations suggest that an oral BPC-157 formulation is technically feasible only via the gastric-window absorption-enhancer route or via local-acting mucosal formulations, with conventional uncoated oral preparations unlikely to achieve clinically relevant systemic exposure. We develop these formulation implications in Section 6.3.

### 5.3. Parenteral Administration: Pharmacokinetic Considerations and Formulation Requirements

Parenteral administration—including intravenous, subcutaneous, and intramuscular routes—bypasses the gastrointestinal absorption barriers and represents the most pharmacokinetically reliable delivery route for BPC-157, as for most peptide therapeutics [9,13,16]. The only human pharmacokinetic data available for BPC-157 were obtained following intravenous administration, confirming systemic exposure with a half-life of less than 30 min [8]. Subcutaneous administration, which is the preferred parenteral route for chronic peptide therapies due to its self-administration compatibility, has not been characterized pharmacokinetically for BPC-157 in humans; intramuscular pharmacokinetic data from He and colleagues [21] demonstrate absolute bioavailability of 14–19% in rats and 45–51% in dogs, but direct subcutaneous characterization—which may differ substantially from IM due to differences in local tissue vascularity and lymphatic uptake—remains absent from the published dataset [13,17,21].

For parenteral formulation development, BPC-157 presents requirements typical of small therapeutic peptides but currently unmet by any published formulation work. Parenteral peptide formulations must satisfy stringent requirements for sterility, particulate matter, pH, osmolality, and chemical and physical stability under storage conditions [6,14]. Peptide stability in aqueous parenteral formulations is frequently compromised by hydrolysis, oxidation, deamidation, aggregation, and adsorption to container surfaces—degradation pathways whose relevance to BPC-157 has not been investigated [14,17]. Forced degradation studies under ICH Q1A stress conditions—acid, base, oxidative, thermal, and photolytic—are a standard requirement for characterizing the degradation profile of a peptide drug substance and identifying the critical quality attributes that formulation development must protect [17]. No such studies have been published for BPC-157.

The short systemic half-life of BPC-157 following intravenous administration raises important questions about the adequacy of conventional immediate-release parenteral formulations for achieving sustained therapeutic exposure [10,18]. If the PK/PD disconnect discussed in Section 4.3 reflects transcriptional or indirect biological mechanisms, intermittent subcutaneous dosing with an immediate-release formulation may provide adequate pharmacodynamic coverage despite rapid plasma clearance. If sustained plasma exposure is required, modified-release parenteral strategies—including depot formulations using biodegradable polymer microspheres, in situ forming gels, or pegylated analogs—would need to be considered, each carrying its own manufacturing complexity and regulatory requirements [39,40].

### 5.4. Topical and Local Administration: Niche Opportunities

Topical and local administration routes represent potentially underexplored opportunities for BPC-157, particularly for musculoskeletal and dermatological indications where direct delivery to the target tissue can circumvent systemic pharmacokinetic limitations [6,7]. Preclinical evidence supports biological activity following topical application to wound sites and intra-articular injection in joint pathology models [1,7]. Local administration strategies offer the theoretical advantage of achieving high local tissue concentrations at the site of injury while minimizing systemic exposure—a profile that would simultaneously address the permeability problem for oral delivery and the rapid systemic clearance problem for parenteral delivery [6,17].

For intra-articular delivery, which has been explored in the single published human clinical observation [7], the synovial joint represents a relatively contained pharmacokinetic compartment with slower clearance than the systemic circulation, potentially extending the residence time of BPC-157 at the target tissue beyond what would be achievable through systemic administration [6,7]. However, no formal intra-articular pharmacokinetic characterization has been performed, and synovial fluid concentrations following intra-articular injection have not been measured. For dermal and transdermal delivery, the molecular weight of BPC-157 at approximately 1419 Da substantially exceeds the empirical 500 Da threshold for passive transdermal permeation, suggesting that conventional transdermal formulations would be ineffective without permeation enhancement strategies such as microneedle arrays, sonophoresis, or chemical enhancers [6,17].

Intranasal administration represents an additional route warranting consideration for CNS indications, given the direct olfactory and trigeminal pathways connecting the nasal mucosa to the central nervous system and the potential to achieve meaningful CNS exposure without systemic administration or blood–brain barrier penetration [17]. No preclinical or clinical data exist for intranasal BPC-157, but the route merits investigation given the CNS pharmacodynamic profile documented in rodent models [2].

### 5.5. Comparative Positioning Among Peptide Therapeutics

Contextualizing BPC-157 within the broader landscape of approved and investigational peptide therapeutics provides a useful reference frame for assessing its development prospects. The global peptide therapeutics market has expanded substantially over the past two decades, with over 80 peptide drugs currently approved by major regulatory agencies and a pipeline of over 150 peptide candidates in active clinical development [13,14,42]. Approved peptide therapeutics span a wide range of molecular sizes, structural classes, and administration routes, and their development trajectories offer instructive precedents for BPC-157 [13,16,20,44].

Structurally, BPC-157 most closely resembles short linear peptides such as oxytocin, desmopressin, and terlipressin in terms of molecular weight and linear backbone architecture, though it lacks the disulfide bridges that provide conformational constraint in many of these compounds [14,16]. Unlike GLP-1 analogs such as semaglutide and liraglutide, which achieve extended half-lives through fatty acid lipidation enabling albumin binding, BPC-157 carries no half-life extension modification, predicting rapid renal and proteolytic clearance consistent with the observed human data [39,40]. Unlike cyclic peptides such as cyclosporine or the RGD-based integrin antagonists, BPC-157 lacks the backbone rigidity and lipophilicity that facilitate membrane permeability in cyclic scaffolds [8,43].

This comparative analysis suggests that BPC-157, in its current unmodified form, occupies a pharmacokinetically challenging position: it is too large for reliable passive absorption, too linear and hydrophilic for membrane permeability, and too rapidly cleared for convenient dosing intervals without formulation intervention. These are not insurmountable obstacles—they are the standard challenges of peptide drug development, and they have been overcome for numerous approved agents through a combination of chemical optimization and formulation science [3,13,20,45]. However, they define a substantial and specific pharmaceutical development agenda that has not yet been initiated for BPC-157. Table 2 presents a comparative overview of BPC-157’s biopharmaceutical properties relative to selected approved peptide therapeutics, illustrating the developmental gaps that distinguish it from compounds that have successfully navigated the pharmaceutical development process.

### 5.6. Therapeutic-Class Positioning: BPC-157 Versus Established Regenerative and Anti-Inflammatory Agents

The pharmacokinetic and physicochemical comparison presented in Table 1 frames BPC-157 within the peptide-therapeutics landscape, but the more clinically relevant question—what would BPC-157 displace, complement, or supersede in the indications it targets?—requires comparison with established therapies for the same conditions. We consider here three therapeutic classes most directly relevant to BPC-157’s indication landscape: recombinant growth factors, anti-inflammatory agents, and regenerative biologics.

Recombinant growth factors. BPC-157’s most consistent preclinical claims involve angiogenesis, fibroblast activation, and tendon, ligament, and muscle regeneration [5,39]—domains that overlap directly with the established clinical use of recombinant human BMP-2 (Infuse Bone Graft^®^ for spinal fusion), PDGF-BB (Regranex^®^ for diabetic foot ulcers), and the historical clinical investigation of VEGF for therapeutic angiogenesis. The mechanistic distinction is substantive: BMP-2, PDGF, and VEGF are large recombinant proteins (≥18 kDa) that bind defined high-affinity tyrosine-kinase or serine/threonine-kinase receptors and act as direct pathway agonists [13,44]. BPC-157, at 1.4 kDa with no identified receptor, is hypothesised to act via indirect modulation—Egr-1/NAB2 transcriptional priming, NO-system regulation, growth hormone receptor upregulation [2,26,27]—which would be expected to produce milder, more pleiotropic, and self-limiting effects. In principle this should translate into a more favourable therapeutic-window profile and reduced risk of the heterotopic ossification, oedema, and oncogenic-signal concerns that have constrained recombinant growth-factor use [44]. The corollary is that BPC-157 is unlikely to match the magnitude of effect achievable with site-directed high-dose growth-factor therapy in indications where defined-pathway maximal stimulation is required (e.g., interbody spinal fusion). Its pharmaceutical advantage is the absence of biologic-class manufacturing complexity (no recombinant cell-line, no protein folding, no glycan characterisation), which would translate into substantially lower cost of goods if the development barriers identified in this review are overcome.

Anti-inflammatory agents. For indications where the BPC-157 evidence base is strongest in mucosal protection and gastrointestinal repair—NSAID enteropathy, inflammatory bowel disease, intestinal anastomosis [46,47,48]—the standard-of-care comparators are NSAID withdrawal, mesalazine, corticosteroids, and anti-TNFα biologics (infliximab, adalimumab) for IBD. The mechanistic positioning is fundamentally different: anti-inflammatory agents suppress an active inflammatory programme, whereas BPC-157 is hypothesised to promote cytoprotection and tissue repair without immunosuppression [1,2]. This is a strategically important distinction because the anti-TNFα class—despite proven IBD efficacy—carries infection, malignancy, and immunogenicity risks that limit long-term tolerability and exclude substantial patient subpopulations. A regenerative cytoprotective agent without immunosuppressive activity would address an unmet need in IBD maintenance therapy, particularly for patients in whom anti-TNFα is contraindicated or has lost efficacy. The historical PL-14736 Phase II data in ulcerative colitis [1,2] support tolerability in this context. The most directly informative regulatory-pathway comparator is teduglutide (a GLP-2 analogue approved for short bowel syndrome), which occupies the regenerative-rather-than-anti-inflammatory niche and demonstrates that the regulatory pathway for such agents is established.

Regenerative biologics. In musculoskeletal indications—the largest preclinical evidence base for BPC-157 [7,49,50]—the established and emerging comparators are platelet-rich plasma (PRP), mesenchymal stromal cell (MSC) preparations, and growth-factor-cocktail injectables. These approaches share with BPC-157 the regenerative-rather-than-symptomatic mechanistic stance, but differ in three ways relevant to pharmaceutical development. First, PRP and MSC products are autologous or minimally manipulated, exempt in many jurisdictions from full drug-product regulatory pathways but correspondingly variable in composition and outcome [44]. BPC-157, as a synthetic peptide of defined sequence, would be regulated as a conventional drug product with full quality-by-design specification—a regulatory burden but also a reproducibility advantage. Second, PRP and MSC products are exclusively local-injection therapies; BPC-157’s multi-route activity, if confirmed in humans, would offer route-of-administration flexibility unavailable to cell-based or autologous-blood-derived products. Third, the cost-of-goods structure differs substantially: peptide synthesis at clinical scale is cheaper per dose than autologous cellular processing or proprietary PRP centrifugation systems, particularly for chronic dosing regimens. The differential is therefore that BPC-157, if developed, would not displace PRP or MSC for single-injection acute musculoskeletal indications where those modalities are entrenched, but would compete strongly in chronic or repeat-dose contexts (tendinopathy, osteoarthritis maintenance) where cellular and autologous strategies are economically and logistically constrained.

Synthesis and implications for indication prioritisation. The therapeutic-class comparison reinforces, rather than alters, the indication prioritisation developed in Section 9.5. The strongest competitive position for BPC-157 lies in indications where (i) existing therapies carry significant tolerability or compliance burdens (chronic IBD, chronic tendinopathy), (ii) regenerative-rather-than-anti-inflammatory mechanism is therapeutically desirable, and (iii) the route-of-administration flexibility of a small synthetic peptide offers logistical or economic advantage over biologic or cell-based comparators. Conversely, BPC-157 is unlikely to displace established therapies in indications where high-magnitude pathway-specific stimulation is required (e.g., recombinant growth factors for spinal fusion) or where existing low-cost generics (NSAIDs, corticosteroids) achieve adequate symptom control with established safety profiles. Table 3 summarises this comparative positioning across the principal therapeutic classes.

## 6. Formulation Challenges and Development Opportunities

### 6.1. The Absence of a Standardized Pharmaceutical Formulation: Implications and Consequences

No pharmaceutical-grade formulation of BPC-157 has been developed, validated, or submitted for regulatory review in any jurisdiction [1,10,11]. The preparations currently available through compounding pharmacies, gray-market suppliers, and research chemical vendors exist outside any quality assurance framework and have not been subjected to the analytical characterization, stability testing, or manufacturing controls required of investigational medicinal products under current Good Manufacturing Practice (cGMP) regulations [11,14]. This absence of formulation standardization has consequences that extend beyond regulatory compliance: without a defined and reproducible pharmaceutical preparation, the dose actually delivered to subjects in off-label or research settings cannot be reliably determined, confounding any attempt to interpret clinical observations or establish dose–response relationships [10,17].

The pharmaceutical development of a peptide drug substance into a viable drug product requires a structured, sequential program addressing drug substance characterization, excipient compatibility, formulation design, analytical method development, stability testing under ICH Q1A conditions, and process development for scalable manufacturing [6,14,17]. For BPC-157, none of these development activities have been reported in the peer-reviewed literature, and no patent literature describes a validated pharmaceutical formulation with defined composition, manufacturing process, and stability data [1]. This represents a gap that is qualitatively different from the clinical evidence gap—it is not merely that human trials have not been conducted, but that the pharmaceutical foundation necessary to conduct trials in a scientifically and regulatorily credible manner has not been established.

### 6.2. Stability Considerations in Pharmaceutical Formulation

BPC-157’s primary sequence (GEPPPGKPADDAGLV) supports a focused stability risk inventory in advance of formal stress-testing data. Three sequence-specific liabilities warrant explicit consideration. First, the Asp10–Asp11–Ala12–Gly13 stretch contains an Asp–Gly junction (positions 11–13, Asp11-Ala12-Gly13, or alternatively read across positions 10–13) that is a recognised locus of aspartyl-bond hydrolysis and succinimide formation under mildly acidic and neutral conditions [14,17]. This is the principal hydrolytic risk in the BPC-157 sequence and the locus that any forced-degradation programme should monitor most closely. Second, the lysine residue at position 7 introduces a glycation liability in the presence of reducing-sugar excipients commonly used in lyophilised peptide formulations, constraining excipient selection toward non-reducing alternatives such as trehalose or mannitol [14]. Third—and as a notable absence—the BPC-157 sequence contains no methionine, tryptophan, or cysteine residues, eliminating the principal oxidative-degradation pathways that drive most peptide formulation development; the sequence likewise lacks asparagine and glutamine, eliminating deamidation as a chemical-degradation concern [14]. The net oxidative and deamidation profile of BPC-157 is therefore unusually favourable among linear peptide drug candidates, a fact that has not been highlighted in the existing literature and that materially simplifies excipient and packaging selection.

Among physical degradation pathways, adsorption to container surfaces is the most pharmaceutically consequential risk for BPC-157 specifically. At the nanogram-to-microgram dose ranges reported in preclinical efficacy studies, surface losses to glass or polymer container materials can represent a clinically relevant fraction of the administered dose, yet this has not been quantified for BPC-157 [14,17]. Fibrillation—the dominant aggregation pathway for many therapeutic peptides—is structurally attenuated in BPC-157 by the three consecutive proline residues at positions 3–5 (GEPPPGKPADDAGLV), which impose conformational rigidity that disfavours β-sheet nucleation; this represents a structural advantage that has not been highlighted in the existing literature. Aggregation under the thermal and shear stresses of manufacturing, however, remains uncharacterised and cannot be excluded on sequence grounds alone.

These sequence-derived predictions establish testable hypotheses but do not substitute for experimental data. Forced-degradation studies under ICH Q1A stress conditions—acid hydrolysis (with particular attention to Asp–Gly cleavage), base hydrolysis, oxidative stress (predicted minimal), thermal stress, and photolytic stress—remain a foundational requirement for any BPC-157 formulation development programme and have not been reported [17].

### 6.3. Oral Formulation Strategies: Opportunities and Feasibility Assessment

The BPC-157-specific delivery literature is, to our knowledge, restricted to patent disclosures and the routes of administration adopted in preclinical efficacy studies; no peer-reviewed pharmaceutical development paper describing a characterised oral formulation of BPC-157 has been published. The Józwiak et al. patent review [1] identifies a Chinese patent describing BPC-157 incorporated into medical dressings for scar repair, in which the peptide content was shown to modulate the bacteriostatic properties of the dressing and where co-formulation with allopurinol was reported to enhance the protective barrier on the wound surface and increase skin cuticle water content [1]. A second patent describes BPC-157 as a component of topical preparations for pain management and injury rehabilitation [1]. Both are topical/local applications; neither describes characterised systemic-delivery formulations, neither provides bioavailability data, and neither has progressed to peer-reviewed pharmaceutical literature with stability, release, or pharmacokinetic characterisation. The implication is that proposals for BPC-157 oral formulation must be derived by analogy with peptides of comparable size and physicochemistry rather than from compound-specific precedent. We assess below the principal candidate strategies, ranked by feasibility for BPC-157 specifically, with selection criteria that include compatibility with the gastric-stability profile, manufacturability at the doses observed in preclinical work (microgram range), regulatory precedent in approved peptide products, and alignment with the tentative BCS class III hypothesis (high solubility, low permeability).

Highest feasibility: gastric-window absorption-enhancer co-formulation. BPC-157 satisfies intrinsically the prerequisite that SNAC co-formulation had to engineer for semaglutide: stability in the gastric environment [1,2,3,20]. The three unknowns specific to BPC-157 that must be resolved before this strategy can progress are: (i) whether gastric mucosal permeability to BPC-157 is measurable in the presence of SNAC or sodium caprate, assessed in ex vivo gastric sac or Ussing chamber models; (ii) whether the polyproline-helix secondary structure of BPC-157 is preserved under the local pH conditions SNAC creates; and (iii) what dose escalation factor is required relative to subcutaneous administration to achieve equivalent systemic exposure. None of these has been reported.

High feasibility: mucoadhesive buccal/sublingual delivery. The buccal mucosa is more permeable to small hydrophilic peptides than the intestinal epithelium, and the route bypasses both intestinal proteolysis and hepatic first-pass metabolism [6,17]. Buccal delivery has been clinically realised for oxytocin and desmopressin—peptides of comparable molecular weight to BPC-157—and the BPC-157 sequence is favourable for buccal formulation in two respects: it lacks methionine (eliminating oxidative degradation in mucoadhesive matrices) and lacks asparagine (eliminating deamidation under buccal conditions) [14]. Mucoadhesive polymers including carbopol, hydroxypropyl methylcellulose (HPMC), and polyacrylic acid derivatives can extend mucosal residence to minutes to hours, potentially enabling clinically meaningful absorption [6]. Feasibility caveats: buccal absorption is patient-compliance-sensitive, and the relatively modest dose range proposed for BPC-157 may make it more suitable for sublingual fast-dissolving formulations than for sustained buccal patches.

For BPC-157 specifically, chitosan nanoparticles offer a dual function relevant to its BCS class III profile: mucosal residence extension and paracellular permeability enhancement via transient tight-junction opening [3,19]. The BPC-157-specific unknowns are: encapsulation efficiency at microgram-range doses (which tends to be low for small hydrophilic peptides in chitosan matrices and has not been reported); nanoparticle stability in gastric juice at pH 1.5–2.0 (relevant because BPC-157 formulations may spend time in the stomach before reaching the intestine); and whether chitosan-mediated tight-junction opening is sufficient to drive paracellular transport of a peptide with the molecular dimensions of BPC-157 (~1419 Da). These are directly testable in Caco-2 and ex vivo intestinal sac models.

The process conditions required for PLGA encapsulation—organic solvent exposure, emulsification, and thermal stress—represent an uncharacterised stability risk for BPC-157, and no forced-degradation data exist that would permit prediction of peptide integrity through the manufacturing process [14,39]. Specifically, the Asp–Gly junction at positions 11–13 identified in Section 6.2 as the principal hydrolytic locus in the BPC-157 sequence may be susceptible to accelerated cleavage under the acidic microenvironment that forms within PLGA matrices as the polymer degrades—a risk that is sequence-specific to BPC-157 and that has not been evaluated.

Not feasible without modification: enteric coating. Enteric coating is designed to protect acid-sensitive compounds from the gastric environment and release them in the small intestine [3]. BPC-157’s gastric stability eliminates the need this technology addresses, and enteric protection would be counterproductive if the gastric mucosa proves to be the primary absorption window—as the SNAC-semaglutide precedent suggests it might [20]. We accordingly identify enteric coating as not indicated rather than untested.

Not feasible without active permeation enhancement: passive transdermal patches. The molecular weight of BPC-157 (~1419 Da) substantially exceeds the empirical 500 Da threshold for passive transdermal permeation [6,17]. Reports of transdermal-patch BPC-157 systems achieving “steady-state plasma concentrations” in the public domain do not appear in peer-reviewed literature and should be treated with caution. Transdermal delivery would require active enhancement strategies—microneedle arrays, sonophoresis, iontophoresis, or chemical enhancers [6,17]—none of which has been investigated for BPC-157.

On this basis, we propose that the three most feasible near-term oral and oromucosal strategies for BPC-157 are, in order: (i) gastric-window absorption-enhancer co-formulation, (ii) mucoadhesive buccal or sublingual delivery, and (iii) chitosan-based mucoadhesive nanoparticles. Each can be initiated immediately using available excipient platforms and standard preclinical absorption models (Caco-2, ex vivo intestinal sac, buccal explant systems), and each can be evaluated for proof-of-principle within a 12–18-month formulation development programme. Table 4 summarises the prioritised formulation strategies for BPC-157 across different routes of administration. These recommendations are derived primarily by analogy with approved peptide therapeutics and are intended as a developmental roadmap rather than a definitive pharmaceutical specification. BPC-157-specific experimental validation will be essential prior to implementation. Among the strategies evaluated, gastric-window absorption-enhancer co-formulation (e.g., SNAC-based systems) offers the highest near-term feasibility. This approach uniquely capitalises on BPC-157’s intrinsic stability in human gastric juice—a critical prerequisite that semaglutide (Rybelsus^®^) required SNAC to engineer, yet which BPC-157 already possesses natively [1,2,3,20]. Mucoadhesive buccal/sublingual delivery ranks second, supported by the peptide’s favourable sequence chemistry (notably the absence of methionine, asparagine, and cysteine) and the established clinical precedent of desmopressin and oxytocin, both of comparable molecular weight [6,14]. Chitosan-based nanoparticles represent the third most promising near-term option, offering combined mucoadhesion and transient tight-junction modulation. All remaining approaches—including PLGA microsphere depots, enteric coatings, and passive transdermal patches—are either lower priority pending dedicated stability and compatibility studies or explicitly unsuitable given BPC-157’s physicochemical profile, as detailed in the following subsections.

### 6.4. Parenteral Formulation Strategies: Modified Release and Half-Life Extension

For parenteral administration, BPC-157’s main formulation challenge is its short systemic half-life. This short half-life limits the duration of plasma exposure after a single dose [10,39,40]. Two broad strategies are available: use a modified-release formulation to extend the absorption phase and sustain plasma concentrations, or implement chemical modification to reduce systemic clearance [39,40].

Biodegradable PLGA microsphere technology has enabled modified-release parenteral formulations for peptide therapeutics. Approved products like leuprolide acetate depot (Lupron Depot^®^) and octreotide long-acting release (Sandostatin LAR^®^) exemplify this, achieving sustained plasma concentrations for weeks to months from one injection [39,40]. Applying similar technology to BPC-157 would involve encapsulating the peptide within PLGA microspheres of defined size and polymer composition. The in vitro release kinetics would be engineered to match the desired pharmacokinetic profile [39]. This approach’s feasibility depends on BPC-157’s compatibility with the PLGA encapsulation process—which includes organic solvent exposure, emulsification, and thermal stress—and on peptide stability within the polymer matrix during storage. Neither aspect has been investigated [14,39].

In situ forming gel systems offer an alternative to microsphere technology. These gels are based on temperature- or pH-sensitive polymers. They form a subcutaneous depot when injected and have been used in peptide therapeutics at late-stage development [40]. These systems are simpler to manufacture than microsphere formulations. They can be designed to provide release profiles from days to weeks, depending on polymer composition and concentration [40].

Half-life extension can be achieved via chemical modification strategies such as PEGylation, fatty acid lipidation, albumin fusion, and Fc fusion. These approaches have improved the clinical utility of many peptide therapeutics by reducing renal clearance and proteolytic degradation [39,40]. For BPC-157, chemical modification would create a new chemical entity different from the original peptide. This would require an entirely new development program and might alter the biological activity profile described in decades of preclinical research [9,13,39]. Whether the benefits of half-life extension outweigh the costs and risks of a new chemical entity program is a strategic question. It cannot be answered until the clinical utility of unmodified BPC-157 is established in controlled human trials [9,13].

### 6.5. Formulation Considerations for Local and Topical Delivery

For local administration routes—including intra-articular injection, topical wound application, and intravesical instillation—the formulation requirements are different from those of systemic delivery. The focus is on local tolerability, sterility, and controlled release at the application site, rather than on optimizing systemic pharmacokinetics [6,7].

Intra-articular formulations for BPC-157 must be sterile, isotonic aqueous preparations. These should be compatible with the synovial environment, and the pH and osmolality must match synovial fluid [4]. Adding viscosity-modifying agents like hyaluronic acid could extend synovial residence time. Increased viscosity reduces drainage through the synovial lymphatics, possibly prolonging local exposure versus a simple aqueous solution [6,40]. Hydrogel-based formulations further extend this concept. They provide a sustained-release depot in the joint space, delivering BPC-157 over days to weeks from one injection [40].

Hydrogel and film-forming formulations with BPC-157 could provide sustained delivery to wound beds while supporting a moist wound environment [6,25]. The compatibility of BPC-157 with hydrogel matrices must be characterized, including its stability during manufacturing, storage, and release. Skin permeation data are also needed to determine whether the peptide stays at the wound surface or penetrates deeper tissue compartments [6,17].

### 6.6. Analytical Development Requirements

Pharmaceutical formulation development for BPC-157 requires parallel development of validated analytical methods for substance and product characterization [14,17]. Necessary analytical capabilities include confirming identity by amino acid analysis and mass spectrometry. Purity is assessed by reverse-phase high-performance liquid chromatography (RP-HPLC) with ultraviolet or charged aerosol detection. Quantification of related substances and degradation products, and potency assessment are also needed [14,17]. No validated in vitro bioassay exists for BPC-157’s biological potency. This reflects the lack of a defined receptor and complicates potency specification for drug product release testing [1,14]. A new bioassay, possibly using a cellular endpoint such as VEGF-stimulated endothelial cell proliferation or Egr-1 reporter gene activation, would be needed for comprehensive analytical development [14,22].

Bioanalytical methods for quantifying BPC-157 in plasma, urine, and tissue homogenates are also needed, as discussed in Section 4.2. LC-MS/MS-based methods provide the necessary selectivity and sensitivity for peptide bioanalysis at nanomolar to picomolar concentrations after dosing. Method validation according to FDA and EMA bioanalytical guidance is a prerequisite for pharmacokinetic data submission to regulatory agencies [17,18].

## 7. Preclinical Safety and Clinical Evidence

### 7.1. Preclinical Safety Profile: Strengths and Limitations

The preclinical safety profile of BPC-157 is unusually favorable by peptide drug candidate standards. Toxicity signals have not been observed across a broad dose range or with multiple administration routes [1,2]. In rodent models, no lethal dose has been established across any route tested. Limit toxicity studies—administering animals the maximum feasible dose, typically 2 g/kg in rodents—showed no clinically meaningful organ-level toxicity. This was determined by histopathology, hematological analysis, or markers of hepatic and renal function [1,2,3,6,7]. The absence of dose-limiting toxicity across a range well above the putative therapeutic range is encouraging. This profile compares favorably with many peptide therapeutics, which often show on-target or off-target toxicity at clinically relevant doses, limiting development [9,13].

The preclinical safety dataset for BPC-157 should be measured against current standards for investigational medicinal product safety. By these standards, it remains substantially incomplete [11,17]. An exception is the repeat-dose intramuscular toxicity study conducted alongside the ADME characterization by He and colleagues [21]. This study showed no visual toxicity or sex-related pharmacokinetic differences after seven days of dosing in both species at therapeutic doses. However, it was not conducted under full GLP conditions required for IND submission. Therefore, it does not qualify as a regulatory-grade toxicology study [11,21]. Regulatory guidance for first-in-human peptide studies requires a defined toxicology package. This must include repeat-dose toxicity studies in two species of appropriate duration, genotoxicity assessment, safety pharmacology studies for cardiovascular, respiratory, and CNS effects, and local tolerance studies for the intended route [11,17]. None of these studies have been reported for BPC-157 in a way that would meet regulatory requirements for IND submission. Described toxicology studies are mostly acute or short-term, conducted as secondary endpoints within efficacy studies. Regulatory agencies do not accept this design as a substitute for dedicated, guideline-compliant toxicology studies [11].

Repeat-dose toxicity data are especially important for BPC-157. The indications of preclinical promise—inflammatory bowel disease, tendinopathy, spinal cord injury—require chronic or subchronic clinical treatment, implying repeated exposure over weeks to months [1,7]. The safety implications of repeated BPC-157 exposure have not been systematically analyzed. This includes possible tachyphylaxis, receptor downregulation, or cumulative organ effects [1,10]. There is also an absence of reproductive and developmental toxicity data, which are necessary for any compound intended for reproductive-age patients [11].

Genotoxicity assessment is important given BPC-157’s pro-angiogenic mechanism. VEGFR2 activation and sustained angiogenic signaling are linked to tumor vascularization in oncological contexts [22,41]. BPC-157’s angiogenic effects seem context-dependent and resolution-oriented instead of constantly active [1,2]. Still, its genotoxic and clastogenic potential has not been evaluated in standard Ames test or chromosomal aberration assays [11]. The absence of these data does not suggest genotoxic liability, but it leaves a regulatory gap. This gap must be addressed before first-in-human studies can be justified.

Safety pharmacology studies evaluating the hERG channel binding potential of BPC-157 have not been reported [11,17]. This standard screen checks for QT-prolongation liability. Peptides are usually considered low risk for hERG inhibition due to their size and charge. Still, regulatory guidance requires explicit evaluation. The clinical study by Lee and Burgess [8] reports no ECG abnormalities in two subjects. However, this does not replace the need for a formal in vitro hERG assay.

### 7.2. Theoretical Safety Considerations: Pro-Angiogenic and Neuropharmacological Risks

Beyond the gaps in formal toxicology characterization, several mechanism-based theoretical safety concerns warrant systematic consideration in the context of pharmaceutical development planning [1,2,10].

The pro-angiogenic properties of BPC-157 involve VEGFR2 activation and VEGF upregulation [22,23]. These properties raise a theoretical concern: BPC-157 could facilitate tumor vascularization in subjects with hidden or existing malignancy. Angiogenesis is a known hallmark of cancer progression. Exogenous pro-angiogenic stimulation is a theoretical oncological risk for any compound acting through VEGF-related pathways [22,41]. Some supporters argue that BPC-157’s angiogenic effects are cytoprotective and resolution-oriented rather than constantly stimulatory. They present corneal angiogenesis data suggesting BPC-157 does not promote pathological neovascularization in that model [2]. However, these arguments are challenged by independent researchers. They note that direct VEGF and NO measurements after BPC-157 administration have not been systematically performed. Also, they emphasize that pro-angiogenic signaling at sufficient concentrations cannot be ruled out with current data [1]. Long-term carcinogenicity studies are required under ICH S1 guidance for compounds meant for chronic human administration. These studies have not been conducted for BPC-157. This gap is a major theoretical safety uncertainty for its clinical development [11].

BPC-157 modulates multiple central neurotransmitter pathways even when administered peripherally [2]. This means it could cause drug interactions with centrally acting medications, but these interactions have not been studied in preclinical or clinical settings. Patients with inflammatory bowel disease—one of BPC-157’s most relevant potential indications—often receive medications such as corticosteroids, immunomodulators, biological agents, and psychiatric drugs due to high rates of anxiety and depression [33,51]. Possible interactions include pharmacodynamic interaction with SSRIs via serotonergic mechanisms [2], with antipsychotics via dopaminergic mechanisms [2], or with benzodiazepines through GABAergic mechanisms [2]. These possibilities have not been investigated and cannot be predicted from current data. Formal drug interaction studies, both in vitro and in vivo, are needed for a thorough clinical development program.

The short half-life of BPC-157 and apparently absent high-affinity receptor binding [1,2] make it hard to predict drug interactions through standard pharmacokinetic mechanisms. Conventional mechanisms like enzyme induction or inhibition and transporter competition rely on sustained drug exposure and well-characterized metabolic pathways. BPC-157’s metabolic pathways are poorly understood [17,18]. Whether BPC-157 is a substrate, inhibitor, or inducer of cytochrome P450 enzymes or relevant drug transporters remains entirely unknown.

### 7.3. Clinical Evidence: A Critical Appraisal

The clinical evidence for BPC-157 comes from three published human studies with a total of fewer than 30 subjects. None were designed as randomized controlled trials [7,10,52]. This evidence base does not support evidence-based clinical recommendations for any indication. It is presented here only to describe what is currently known about human exposure to BPC-157. Table 5 summarizes the three published studies.

Lee and Burgess [10] conducted a pharmacokinetic and safety pilot study. Two healthy adult volunteers received intravenous BPC-157 at 10 mg and 20 mg on consecutive days. Section 4.2 discusses this study as the only human pharmacokinetic data for BPC-157. This study has critical limitations: an extremely small sample size, a specific age range of subjects, a lack of oral or subcutaneous pharmacokinetic data, and incomplete reporting of bioanalytical methods. No adverse events were reported in the two subjects across both doses. While this is positive, it is not statistically meaningful; a sample size of two provides about 86% confidence of detecting only those adverse events with a true incidence of 10% or higher. Rare and uncommon adverse events remain entirely undetectable [10,17].

The retrospective clinical observation by Vasireddi and colleagues [7] examined 16 patients with chronic knee pain who received intra-articular BPC-157 injections and reported that 87.5% of patients experienced significant pain relief at 6 to 12-month follow-up. While this observation is clinically interesting, the study design—retrospective, uncontrolled, without a standardized pain assessment instrument, blinding, or placebo comparison—precludes any causal inference and is subject to substantial recall bias, selection bias, and regression to the mean [7]. The high placebo response rate documented in intra-articular injection trials for knee pain—frequently exceeding 40–50% in randomized controlled trials—renders uncontrolled observations in this indication particularly difficult to interpret [7].

The pilot study by Lee, Walker, and Ayadi [52] investigated intravesical BPC-157 administration in 12 patients with interstitial cystitis and reported 80–100% symptom resolution. Interstitial cystitis is characterized by high symptom variability and substantial placebo response, and the absence of a control arm makes it impossible to distinguish drug effect from natural disease fluctuation or placebo response in this study [52]. Furthermore, the intravesical route represents a specialized local delivery modality whose pharmacokinetic and pharmacodynamic characteristics differ substantially from systemic administration routes, limiting the generalizability of these outcomes to other indications [6,53].

Across all three studies, no serious adverse events were reported, which is reassuring but statistically uninformative given the total sample size and study durations [7,10,52]. The cumulative human exposure to BPC-157 documented in the peer-reviewed literature is insufficient to characterize the safety profile of the compound with any statistical confidence, and the absence of reported adverse events in fewer than 30 subjects cannot be interpreted as evidence of safety in the pharmacological sense [10,17].

### 7.4. The Evidence Hierarchy and Its Implications

The totality of available clinical evidence for BPC-157— three uncontrolled pilot studies with a combined enrollment of fewer than 30 subjects—places it at Level IV to V on standard evidence hierarchies, below the threshold required to support clinical practice recommendations under any major evidence-based medicine framework [7,10,52]. The 2025 systematic review by Vasireddi and colleagues identified a ratio of approximately 544 preclinical studies to one qualifying clinical study in the BPC-157 literature [7]—a disproportion that is without precedent among peptide compounds at a comparable stage of public and clinical interest.

This evidentiary situation has a specific regulatory consequence: BPC-157 cannot currently be evaluated for clinical benefit or harm with any statistical confidence, meaning that neither its adoption into clinical practice nor its definitive rejection as a therapeutic candidate is scientifically justified on the basis of available human data [10,11]. The appropriate response to this situation is not continued off-label use pending spontaneous evidence accumulation, but the initiation of structured clinical investigation under institutional oversight, beginning with the pharmacokinetic and safety characterization program outlined in Section 4.5 and progressing to randomized controlled efficacy trials in well-defined indications [11,13,17].

## 8. Regulatory Landscape and Translational Development Barriers

### 8.1. Current Regulatory Status: A Comparative Overview

The regulatory status of BPC-157 across major pharmaceutical jurisdictions shows a clear pattern. The compound has drawn enough attention to prompt formal regulatory evaluation. Yet, it lacks the evidence needed for investigational or approved use under any major regulatory framework [11]. Recognizing the specific regulatory designations of BPC-157 and their practical implications is essential to define a realistic translational pathway.

In the United States, BPC-157’s regulatory status under Section 503A of the Federal Food, Drug, and Cosmetic Act has changed significantly. Previously, the FDA classified BPC-157 as a Category 2 bulk drug substance. This meant the substance posed safety concerns or lacked sufficient clinical utility to justify compounding for patients [11]. As of 15 April 2026, the FDA removed BPC-157 from Category 2 because the original nominators withdrew their nominations [11]. This removal only follows the withdrawal and is not a regulatory decision on safety or efficacy. The FDA plans to consult the Pharmacy Compounding Advisory Committee (PCAC) on 23 July 2026. This consultation will consider adding BPC-157 acetate and BPC-157 (free base) to the 503A bulks list, specifically for ulcerative colitis [11]. This represents a shift for BPC-157: it moves from being administratively prohibited to actively reviewed for potential compounding use. This depends on the PCAC consultation and later FDA rulemaking. Clinical implications are substantial. A favorable PCAC recommendation could reopen compounding for BPC-157 in the US and create a structured regulatory dialogue, which has been missing since NCT02637284 ended. Developers and investigators should closely watch the PCAC proceedings. The outcome will impact regulatory strategy and the commercial potential of any BPC-157 program.

The European Medicines Agency (EMA) has not issued a regulatory opinion on BPC-157. The compound falls under the EMA’s general rules for investigational medicinal products. A Clinical Trial Authorization (CTA) and an Investigational Medicinal Product Dossier (IMPD) must be approved before clinical study in the EU [11,17]. Requirements for a peptide IMPD include drug substance characterization, product composition and manufacture, non-clinical pharmacology and toxicology, and clinical data. These require documentation beyond what current BPC-157 evidence provides [11,17]. Specifically, BPC-157 lacks a well-defined, pharmaceutical-grade drug substance with characterized identity, purity, and stability. There also is no complete nonclinical toxicology package as outlined in Section 7.1. These gaps prevent IMPD approval under current EMA guidance [11].

The World Anti-Doping Agency (WADA) temporarily placed BPC-157 on the 2022 Prohibited List under S0 Unapproved Substances. This decision was later reversed. The S0 category includes substances that lack regulatory approval and may pose risks to athlete health [12]. WADA’s reversal was not a finding on safety or efficacy. Instead, it reflected the difficulty of banning a substance that cannot be reliably detected or analyzed in doping controls [12]. This WADA case highlights widespread regulatory concern about BPC-157. It also highlights the reputational risks of using BPC-157 outside clinical frameworks.

### 8.2. Requirements for an Investigational New Drug Application

A credible IND application for BPC-157 in the United States needs much more data than currently published [11,17]. According to 21 CFR Part 312, an IND must contain three parts. First, it needs enough animal pharmacology and toxicology studies to assess investigation safety. Next, it requires manufacturing information that proves the identity, quality, purity, and strength of the drug. Finally, it must include clinical protocols and investigator details [11].

**Figure 3:**
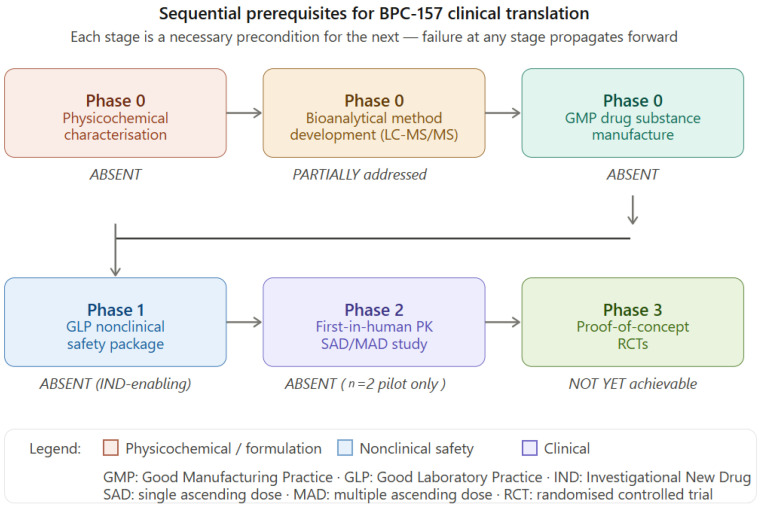
Sequential biopharmaceutical and regulatory barriers limiting BPC-157 clinical translation. Each identified gap represents a necessary precondition for advancement to the subsequent stage; failure to address any single element propagates through the entire development chain and prevents meaningful progress toward clinical investigation. Current status annotated beneath each stage [7,54]. GMP: Good Manufacturing Practice; GLP: Good Laboratory Practice; IND: Investigational New Drug; PK: pharmacokinetics; SAD: single ascending dose; MAD: multiple ascending dose; RCT: randomized controlled trial. Figure created by the authors.

For the animal pharmacology and toxicology section, BPC-157’s available preclinical dataset must be greatly expanded before submitting an IND [11,17]. Missing or incomplete studies include repeat-dose toxicity of at least 28 days in two species (often rat and non-human primate or dog) under Good Laboratory Practice (GLP) conditions. A full safety pharmacology battery is also needed. This includes cardiovascular (with hERG binding and in vivo telemetry), respiratory, and CNS testing under ICH S7A/S7B guidance. Additionally, genotoxicity studies like an Ames test and an in vitro chromosomal aberration or micronucleus assay are required. Local tolerance studies matched to the clinical route must also be conducted [11,17]. This toxicology package typically takes 18 to 36 months and costs several million USD for a peptide candidate. These requirements pose a major hurdle for academic or small-sponsor programs without industry partners [13,44]. Figure 3 shows how each step depends on completion of earlier steps. Ignoring any of these requirements stalls further clinical development.

For manufacturing data, the IND must describe the drug substance synthesis or isolation, as well as set specifications for identity, strength, quality, and purity. It also must outline the drug product’s composition and manufacturing process [11,14]. For BPC-157, solid-phase peptide synthesis (SPPS) using Fmoc chemistry is the standard approach. This synthetic pathway is well documented in the literature [1,14]. However, GMP-compliant manufacturing for BPC-157—including process validation, impurity control, and certification—has not yet been reported. No GMP product suitable for clinical use exists [11,14]. Establishing GMP manufacturing for a peptide drug requires major investments in analytical systems, process development, and quality management. None have been started for BPC-157 [14,17].

### 8.3. The Abandoned Phase I Trial: Lessons and Consequences

The termination without results publication of the Phase I clinical trial registered as NCT02637284—sponsored by PharmaCotherapia and designed to enroll 42 healthy volunteers for safety and pharmacokinetic assessment of BPC-157—represents perhaps the most consequential single event in the translational history of this compound [10,11]. Had this trial been completed and its results published, the field would possess the foundational human pharmacokinetic and safety data that currently constrain every aspect of BPC-157’s development—dose selection, formulation design, route optimization, and regulatory strategy.

The reasons for the trial’s termination have not been publicly disclosed, and the absence of results publication—which would be required under ICMJE standards for any trial conducted after 2005 and is mandated under US law for trials registered on ClinicalTrials.gov—represents a failure of research transparency with ongoing consequences for the field [10,11]. The absence of this dataset has created a decade-long evidence vacuum that has been partially filled by uncontrolled clinical observations and off-label use, generating exposure data that cannot be systematically analyzed and contributing to the information environment that regulators and clinicians must navigate [10,11].

The lessons of NCT02637284 for future development planning are specific. First, industry sponsorship alone is insufficient to ensure trial completion and results dissemination; future clinical investigations of BPC-157 should incorporate data sharing commitments and publication timelines as conditions of institutional and regulatory approval [11]. Second, the pharmacokinetic objectives of NCT02637284—characterizing the human PK profile of BPC-157—remain unmet and constitute the single highest-priority evidence gap for translational development, regardless of therapeutic indication [14,17]. Third, the reinitiating of a Phase I program would require GMP drug substance and drug product manufacture, a comprehensive nonclinical toxicology package, and a validated bioanalytical method—prerequisites that were presumably met for NCT02637284 but whose documentation has not entered the public domain [11,14].

### 8.4. Intellectual Property Landscape and Commercial Development Incentives

The intellectual property landscape surrounding BPC-157 presents both opportunities and barriers for commercial pharmaceutical development [1,44]. Multiple patent applications covering BPC-157 compositions, formulations, and therapeutic uses have been filed by various inventors and organizations over the past three decades, creating a complex freedom-to-operate environment that prospective developers would need to navigate [1]. The original composition-of-matter patents for BPC-157 filed by the University of Zagreb and Pliva in the 1990s have likely expired or are approaching expiration, potentially enabling generic or biosimilar development approaches but simultaneously reducing the commercial exclusivity that typically incentivizes pharmaceutical industry investment in late-stage clinical development [1,44].

The absence of commercial exclusivity protection—or uncertainty about its availability through formulation or method-of-use patents—is a significant disincentive for industry-sponsored Phase II and III development, as the substantial investment required for late-stage clinical trials is typically justified by the expectation of market exclusivity during the period of patent protection [44]. This commercial dynamic may partly explain the paradox of extensive preclinical research conducted primarily by an academic group alongside an almost complete absence of industry-sponsored clinical development—a pattern seen with other academically championed compounds that lack a clear intellectual property pathway to commercial return [13,44].

Orphan drug designation, available in the US for conditions affecting fewer than 200,000 patients and in the EU for conditions affecting fewer than five in 10,000, could provide a regulatory and commercial pathway for BPC-157 development in specific niche indications—such as short bowel syndrome or spinal cord injury—where patient populations are small, unmet need is high, and the orphan drug incentives of seven-year market exclusivity and reduced regulatory fees could improve the commercial viability of a development program [11,44]. Exploration of orphan designation eligibility for one or more BPC-157 indications represents a specific and actionable regulatory strategy that has not been publicly pursued.

### 8.5. Proposed Translational Development Roadmap

Synthesizing the biopharmaceutical, pharmacokinetic, safety, and regulatory considerations discussed across this review, a prioritized translational development roadmap for BPC-157 can be articulated, organized into sequential phases that reflect the logical dependencies between development activities [9,11,13,17].

Phase 0—Pharmaceutical and Analytical Foundation (estimated timeline: 12–18 months). Development and ICH validation of an LC-MS/MS bioanalytical method for BPC-157 quantification in plasma, urine, and tissue matrices; forced degradation studies under ICH Q1A stress conditions to characterize the drug substance degradation profile; physicochemical characterization including solubility profiling across the physiological pH range, preliminary permeability assessment in Caco-2 and PAMPA models, and plasma protein binding determination; development of a GMP-compliant drug substance manufacturing process with full analytical characterization; and development of at least one GMP drug product formulation suitable for parenteral administration in first-in-human studies [14,17,18]. Table 6 presents a prioritized translational development roadmap for BPC-157, organized into sequential phases that reflect the logical dependencies between development activities. Each phase represents a necessary precondition for initiation of the subsequent phase; the roadmap is not proposed as a definitive development plan but as a framework illustrating the minimum scope and sequence of activities required to bring BPC-157 to the point of evidence-based clinical evaluation.

Phase 1—Nonclinical Safety Package (estimated timeline: 18–36 months, partially overlapping with Phase 0). GLP-compliant repeat-dose toxicity studies of 28-day duration in rat and dog; safety pharmacology battery under ICH S7A/S7B including hERG binding, cardiovascular telemetry, respiratory function, and CNS assessment; genotoxicity battery including Ames test and in vitro micronucleus assay; local tolerance studies for the intended clinical route; and preliminary pharmacokinetic characterization in both species including oral and subcutaneous bioavailability assessment [11,15].

Phase 2—First-in-Human Pharmacokinetic and Safety Study (estimated timeline: 12–18 months following Phase 1 completion). Randomized, double-blind, placebo-controlled single ascending dose and multiple ascending dose study in healthy volunteers, adequately powered for pharmacokinetic characterization across a defined dose range, with full ADME assessment including mass balance study, metabolite identification, and renal and hepatic clearance quantification; population pharmacokinetic analysis incorporating relevant covariates; and formal drug interaction assessment with representative co-medications [10,17,18].

Phase 3—Indication-Specific Proof-of-Concept Studies. Randomized, placebo-controlled Phase IIa studies in two to three well-defined indications selected on the basis of preclinical evidence strength, unmet clinical need, and feasibility of endpoint assessment—with NSAID-induced enteropathy, Achilles tendinopathy, and inflammatory bowel disease representing the most scientifically justified candidates based on the available preclinical literature [1,7,11].

This roadmap is not proposed as a definitive development plan—which would require sponsor commitment, regulatory agency interaction, and indication-specific clinical feasibility assessment—but as a framework illustrating the sequential nature and approximate scope of the development activities required to bring BPC-157 to the point of evidence-based clinical evaluation [9,13,17].

## 9. Discussion

### 9.1. The Paradox of BPC-157: Pharmacological Richness Versus Pharmaceutical Poverty

BPC-157 has accumulated an unusually broad preclinical evidence base spanning gastrointestinal cytoprotection, musculoskeletal repair, cardiovascular protection, and neurological effects. Independent systematic assessments, most notably the 2025 review by Vasireddi and colleagues published in HSS Journal (Springer Nature), confirm consistent beneficial effects in rodent models of tissue injury while highlighting the near-complete absence of high-quality clinical data [7]. However, the vast majority of primary experimental studies originate from a single research group at the University of Zagreb, with many recent publications appearing in the same cluster of open-access journals. Although this body of work shows internal consistency, the limited independent replication by other laboratories constitutes a significant limitation for translational interpretation [7,53,55,56,57].

### 9.2. The Biopharmaceutical Gap as the Primary Translational Barrier

The central conclusion of this review is that the main obstacle to clinical advancement of BPC-157 is not a lack of biological activity, but the profound deficiency in essential pharmaceutical and regulatory-grade data. As detailed in Section 4, Section 5 and Section 6, critical gaps remain in human pharmacokinetics, validated formulations, stability characterisation, and dose justification [10,21]. These deficiencies render current off-label use scientifically unjustified and preclude rational clinical trial design.

### 9.3. Contextualizing BPC-157 Within the Current Peptide Therapeutics Landscape

When evaluated against contemporary standards in peptide drug development, BPC-157 occupies a challenging but not unprecedented position. Its rapid systemic clearance, documented in the independent ADME study by He and colleagues (Frontiers in Pharmacology, 2022) [21], is consistent with expectations for an unmodified linear pentadecapeptide [18]. Successful peptide therapeutics such as oral semaglutide and teduglutide reached approval only after substantial pharmaceutical optimisation—a stage BPC-157 has not yet entered. Cardiovascular, gastrointestinal, and musculoskeletal findings, while mechanistically interesting, require independent replication and integration with rigorous pharmacokinetic–pharmacodynamic data before they can support clinical development [58,59,60,61].

### 9.4. The Scientific Controversy and Its Implications for Development

Ongoing debate in the literature, particularly the critical commentary by Józwiak and colleagues and the subsequent exchange [1,54,62,63], highlights unresolved questions regarding angiogenesis, nitric oxide signalling, and potential oncological risk. Such controversy is scientifically healthy but highlights the need for independent verification and cautious interpretation in any future development programme.

### 9.5. Indication Selection Strategy: A Biopharmaceutical Perspective

The selection of the first clinical indication for BPC-157 development has implications not only for clinical trial design but for pharmaceutical formulation strategy, as different indications favour different routes of administration and formulation types. A biopharmaceutical perspective on indication selection—which has not previously been applied to BPC-157—yields specific strategic recommendations.

From a biopharmaceutical perspective, indication selection for BPC-157’s first clinical programme is directly coupled to route-of-administration feasibility. Gastrointestinal indications favour oral delivery, which BPC-157’s gastric stability uniquely supports and where local mucosal effects may be sufficient without systemic absorption [1,2,46,64]. This positioning is reinforced by the established regulatory precedent for intestinotrophic peptide therapeutics: teduglutide (a GLP-2 analogue approved for short bowel syndrome) demonstrates that a regenerative rather than immunosuppressive mechanism of action can support regulatory approval in a gastrointestinal indication, and that the clinical development pathway for such agents is well defined [65]. The clinical need in this indication domain has been characterised in authoritative reviews published outside the BPC-157-specific literature [60,61], providing an independent frame of reference for positioning BPC-157 against established therapeutic standards. Musculoskeletal indications favour local parenteral administration, circumventing systemic pharmacokinetic limitations [7,66]; the independent systematic review by Vasireddi and colleagues [7] identifies tendinopathy and ligament injury as the indications with the most extensive and consistent preclinical dataset. Within this domain, the regulatory and clinical development pathway for locally administered investigational peptides in tendinopathy and osteoarthritis has been increasingly characterised, with several peptide and growth-factor candidates having completed Phase I and Phase II evaluation, providing a framework for trial design [67,68]. CNS indications are biopharmaceutically the most challenging and should be considered only after systemic PK parameters are established in humans [2,17,18]. Cardiovascular and vascular indications represent high unmet-need targets appropriate for Phase II development following the pharmacokinetic foundation programme [69,70,71,72,73,74,75], and the clinical significance of these targets is supported by a substantial body of cardiology and vascular medicine literature independent of the BPC-157 preclinical record [58,59]. Table 7 summarises this biopharmaceutical indication prioritisation.

### 9.6. The Regulatory Opportunity: Expedited Pathways

Three expedited regulatory mechanisms warrant active exploration for BPC-157. Fast Track designation is immediately pursuable in indications of high unmet need such as spinal cord injury, short bowel syndrome, or treatment-refractory IBD [1,2,11,46,64]. Breakthrough Therapy designation is not currently achievable without controlled clinical data but becomes accessible following a positive Phase IIa result [11]. Orphan drug designation—carrying seven-year market exclusivity and reduced regulatory fees in the US—is the most commercially actionable near-term pathway and should be explored for spinal cord injury and short bowel syndrome as a priority, given the alignment of BPC-157’s preclinical profile with orphan indication criteria and the commercial development incentives it would provide [11,44].

### 9.7. Limitations of This Review

The present review has several important limitations that should frame the interpretation of its conclusions. First, it is a narrative rather than a systematic review and did not apply a formal risk-of-bias instrument or quantitative meta-analytic synthesis. As such, the weighting of individual studies reflects expert judgment rather than predefined scoring criteria, and publication bias or selective reporting cannot be excluded [53,55,56,57].

Second, the primary BPC-157 evidence base is itself structurally limited. A substantial proportion of preclinical data originates from a single academic group and a relatively narrow network of collaborators, with many studies published in a limited set of journals. This concentration reflects the current structure of the field rather than any editorial preference of the present authors, but it nonetheless constrains the robustness and generalisability of BPC-157-specific findings, as confirmed by the independent systematic assessment of Vasireddi and colleagues [7]. Independent replication in diverse research settings and dissemination across a broader range of journals remain sparse and should be considered a major unmet need [53,55,56,57]. Additionally, the sole formal pharmacokinetic dataset [21] derives from a group whose corresponding author has a disclosed affiliation with a commercial peptide synthesis enterprise; this does not impugn data integrity, but readers should weigh this context when interpreting bioavailability figures and dose-translation proposals.

Third, although Section 9 draws on BPC-157-specific publications, its central arguments are comparator data are distributed across multiple publishers and therapeutic classes, and they were included precisely to mitigate over-reliance on any single cluster of BPC-157 papers and to contextualise BPC-157 within established principles of pharmaceutical science.

Fourth, several key aspects of BPC-157 biopharmaceutics remain incompletely characterised or entirely unreported, including intestinal stability, epithelial permeability, plasma protein binding, and detailed solution structure [17,18]. The formulation and development proposals advanced in this review therefore rely, by necessity, on physicochemical reasoning and analogy with approved peptide therapeutics [3,13,20] rather than on compound-specific experimental data. These proposals should be regarded as a developmental roadmap to be tested, not as a definitive specification.

Finally, regulatory and clinical inferences are constrained by the paucity of human data [10,11]. The available clinical experience comprises small, uncontrolled pilot studies with non-standardised preparations and no validated pharmacokinetic characterisation [10,21]. Until rigorously designed, controlled trials using pharmaceutical-grade formulations are conducted, any extrapolation from preclinical or anecdotal human data to clinical practice must remain cautious [11,17]. The review’s objective is to outline the biopharmaceutical and translational barriers that would need to be addressed, not to advocate for off-label or non-regulated use.

## 10. Conclusions

BPC-157 is a synthetic pentadecapeptide with a pharmacologically compelling but pharmaceutically underdeveloped profile. Preclinical evidence accumulated over three decades across gastrointestinal, musculoskeletal, cardiovascular, and central nervous system models demonstrates consistent cytoprotective and regenerative activity through mechanistically coherent pathways involving VEGFR2-mediated angiogenesis, Egr-1 transcription factor activation, nitric oxide system modulation, and growth hormone receptor upregulation. Its unusual resistance to gastric proteolysis, multi-route biological activity, and absence of dose-limiting toxicity in preclinical models distinguish it from the majority of linear peptide drug candidates.

However, BPC-157 currently lacks the foundational pharmaceutical characterization required for clinical development. No formal solubility profiling, intestinal permeability data, plasma protein binding determination, or solution-phase structural characterization has been published. No pharmaceutical-grade formulation exists. No validated bioanalytical method for quantification in biological matrices has been reported. The preclinical pharmacokinetic profile has been formally characterized in rats and beagle dogs, establishing sub-30-min half-life, linear dose-proportional kinetics, 14–51% intramuscular bioavailability (species-dependent), and urinary/biliary excretion; the only human pharmacokinetic study enrolled two subjects and did not include oral or subcutaneous pharmacokinetic arms, providing preliminary directional confirmation but insufficient data for clinical dose selection. A Phase I trial registered in 2016 (NCT02637284) was terminated without results publication, leaving a critical and persistent evidence gap.

Based on available physicochemical data, a provisional working hypothesis of tentative BCS Class III character (high solubility, low permeability) is offered solely as a framework for guiding future experimental design and formulation strategy; this hypothesis requires validation through standardized regulatory-grade solubility and permeability assays before any classification can be formally proposed. Its principal implication for development strategy is that permeability enhancement rather than dissolution optimization represents the appropriate direction for oral formulation work. The observed pharmacokinetic–pharmacodynamic disconnect—systemic half-life under 30 min contrasting with prolonged biological effects—remains mechanistically uncharacterized and constitutes a primary obstacle to rational dosing regimen design. Formulation approaches warranting systematic investigation include absorption enhancer co-formulation for oral delivery, PLGA microsphere depot systems for parenteral modified release, and hydrogel-based matrices for local musculoskeletal and intra-articular administration.

The nonclinical safety package required for IND submission—including GLP-compliant repeat-dose toxicity studies in two species, a full ICH S7A/S7B safety pharmacology battery, genotoxicity assessment, and long-term carcinogenicity data addressing the theoretical oncological risk of sustained pro-angiogenic signaling—has not been conducted. The regulatory pathway most immediately accessible involves orphan drug designation in high-unmet-need indications such as short bowel syndrome or spinal cord injury, where preclinical evidence is strongest and commercial development incentives are available.

Future research priorities, in order of developmental precedence, are: development and ICH validation of LC-MS/MS bioanalytical methods; physicochemical characterization including BCS-compliant solubility and permeability profiling; GMP drug substance manufacture and drug product development; a GLP nonclinical safety package; and a first-in-human single and multiple ascending dose pharmacokinetic study in an adequately powered healthy volunteer population. Indication-specific proof-of-concept studies in gastrointestinal and musculoskeletal indications, where local delivery strategies can circumvent systemic pharmacokinetic limitations, represent the most biopharmaceutically justified early clinical targets.

The primary barrier to BPC-157’s clinical translation is not its pharmacological profile, which is substantiated, nor its preclinical safety record, which is reassuring, but the absence of the pharmaceutical science infrastructure that is prerequisite to any credible clinical program. Addressing this gap systematically and sequentially represents the minimum necessary investment to determine whether three decades of consistent preclinical evidence can be translated into validated human therapeutic benefit.

## Figures and Tables

**Table 1:** Stability profile of BPC-157 (GEPPPGKPADDAGLV) across pharmacologically and pharmaceutically relevant compartments and degradation pathways. t½: elimination half-life; IV: intravenous; Met: methionine; Cys: cysteine; Trp: tryptophan; Asn: asparagine; Gln: glutamine; Asp: aspartic acid; N/A: not applicable.

Stability Domain	Parameter/Pathway	Status for BPC-157	Mechanistic Basis	Pharmaceutical Implication	References
Gastric stability	Resistance to pepsin and HCl (pH 1–2)	High—intact peptide recoverable after prolonged in vitro incubation	Conformational rigidity of N-terminal polyproline II helix sterically occludes proteolytic recognition sites	Oral delivery targeting the gastric absorption window is mechanistically justified; enteric coating not indicated	[1,2]
Plasma stability	Systemic elimination half-life	Poor—t½ < 30 min (IV: 15.2 min rat, 5.27 min dog; IV: <30 min human pilot)	Rapid renal filtration and systemic proteolytic hydrolysis to six peptide fragments (M1–M6)	Short dosing intervals or modified-release parenteral formulations required for sustained exposure; immediate-release may suffice if PK/PD disconnect reflects indirect mechanisms	[10,21]
Intestinal stability	Resistance to intestinal proteases and brush-border enzymes	Not determined	N/A	Critical uncharacterised gap; must be addressed before oral bioavailability can be predicted or formulation development initiated	—
Hepatic first-pass extraction	Hepatic metabolism foll owing oral absorption	Not determined	N/A	Unknown contribution to oral bioavailability loss; hepatic extraction study required as part of ADME characterisation programme	—
Oxidative degradation	Met oxidation; Cys disulfide formation; Trp oxidation	Minimal risk—sequence contains no Met, Cys, or Trp residues	Absence of oxidation-prone side chains	Antioxidants not required in formulation; packaging oxygen control may be simplified relative to most peptide drug products	[14,17]
Deamidation	Asn → Asp/isoAsp; Gln → Glu conversion	Not applicable—sequence contains no Asn or Gln residues	Absence of deamidation-susceptible residues	Deamidation monitoring not required in stability programme; pH control less critical than for Asn/Gln-containing peptides	[14,17]
Aspartyl-bond hydrolysis	Asp–X peptide bond cleavage; succinimide formation	Primary chemical liability—Asp10–Asp11–Ala12–Gly13 stretch represents principal hydrolytic risk under acidic and neutral conditions	Asp–Gly junction susceptible to succinimide-mediated isomerisation and hydrolysis; accelerated under PLGA matrix acidic microenvironment	Forced degradation programme must monitor Asp10–Asp11 cleavage products; PLGA compatibility requires dedicated assessment; pH optimisation of parenteral formulation critical	[14,17]
Glycation (Maillard reaction)	Lys7 ε-amino group reaction with reducing sugars	Potential liability in the presence of reducing-sugar excipients (e.g., lactose, glucose, maltose)	Lys7 pKa ~10.5; free ε-amino group reactive toward carbonyl groups under typical lyophilisation and storage conditions	Excipient selection must exclude reducing sugars; trehalose or mannitol preferred as lyoprotectants	[14]
Aggregation/fibrillation	β-sheet nucleation and fibril formation	Structural risk attenuated—three consecutive Pro residues at positions 3–5 disfavour β-sheet conformation; thermal/shear aggregation uncharacterised	Polyproline motif imposes conformational rigidity incompatible with β-sheet stacking; does not exclude stress-induced aggregation during manufacturing	Aggregation under manufacturing stresses (shear, freeze–thaw, thermal) must be characterised; SEC and DLS monitoring recommended in stability protocol	[14,17]
Adsorption to container surfaces	Peptide loss via adsorption to glass or polymer surfaces	Not determined; high risk anticipated at microgram-range doses	Low molecular weight and amphipathic character increase surface interaction probability; dose range (ng–µg/kg) amplifies relative impact of surface losses	Container surface adsorption study required; polysorbate 80 or human serum albumin as blocking agents should be evaluated; low-bind polymer vials or siliconised glass recommended	[14,17]

**Table 2:** Physicochemical and biopharmaceutical properties of BPC-157 compared to selected approved peptide therapeutics. MW: molecular weight; BCS: Biopharmaceutical Classification System; SC: subcutaneous; IM: intramuscular; IV: intravenous; SNAC: sodium N-[8-(2-hydroxybenzoyl)amino]caprylate; N/A: not applicable. Data for approved therapeutics from product prescribing information and published pharmacokinetic literature. BCS classification for BPC-157 is tentative, based on physicochemical reasoning; all other BPC-157 values from Lee & Burgess [10] or as reported in the text.

Property	BPC-157	Semaglutide	Cyclosporine	Oxytocin	Desmopressin
MW (Da)	~1419	~4114	~1203	~1007	~1069
Structure	Linear	Linear + lipidation	Cyclic	Cyclic (disulfide)	Cyclic (disulfide)
Gastric stability	High (reported)	Low	Moderate	Low	Low
Oral bioavailability	Unknown	0.4–1% (with SNAC)	~30%	<1%	<1%
Half-life (human)	<30 min IV (human [8]); 15.2 min IV rats, 5.3 min IV dogs [31]	~1 week	~6–24 h	~3–5 min	~75 min
BCS classification	Tentative III	N/A (biologic)	Class II	Class III	Class III
Approved route	None	SC, oral	Oral, IV	IV, IM, intranasal	Oral, SC, intranasal

**Table 3:** Comparative therapeutic positioning of BPC-157 versus established regenerative and anti-inflammatory therapies. The table compares the principal classes of competing or alternative therapies across the indications where BPC-157 has the strongest preclinical evidence base. “Differentiator” identifies the property that would justify BPC-157 development versus the comparator class; “Limiting factor” identifies the property that would constrain it. UC: ulcerative colitis; CD: Crohn’s disease; PRP: platelet-rich plasma; MSC: mesenchymal stromal cell; SC: subcutaneous; IV: intravenous; GH: growth hormone; NO: nitric oxide.

Class	Representative Agents	Mechanism	Route/Status	Differentiator vs. BPC-157	Limiting Factor vs. BPC-157
Recombinant growth factors	BMP-2 (Infuse^®^), PDGF-BB (Regranex^®^), rhVEGF	Direct receptor agonism (TK/Ser-Thr kinase)	Local implant/topical; approved (narrow indications)	Defined-pathway maximal stimulation; established regulatory pathway	Biologic-class manufacturing; heterotopic ossification, oedema, oncogenic-signal concerns
Anti-TNFα biologics	Infliximab, adalimumab	Cytokine neutralisation	IV/SC; approved for IBD, rheumatologic indications	Proven IBD efficacy; established reimbursement	Immunosuppression; infection/malignancy risk; immunogenicity; high cost
5-ASA derivatives	Mesalazine, sulfasalazine	Local mucosal anti-inflammatory	Oral/rectal; approved generic (UC, CD)	First-line tolerability; generic availability	Modest efficacy in moderate-to-severe disease; no regenerative effect
Corticosteroids	Prednisolone, budesonide	Broad immunosuppression	Oral/IV/topical; approved generic	Rapid onset; very low cost	Chronic-use toxicity precludes maintenance therapy; no regenerative effect
Platelet-rich plasma	Autologous PRP preparations	Autologous growth-factor cocktail	Local intra-articular/intra-tendinous; minimally regulated	Autologous safety profile; widespread practitioner familiarity	Variable composition; single-injection logistics; no chronic-use evidence base
Mesenchymal stromal cells	Autologous/allogeneic MSC products	Paracrine immunomodulation; trophic support	Local injection; investigational/regional approvals	Broad regenerative repertoire	Manufacturing complexity; donor variability; regulatory burden
Intestinotrophic peptides	Teduglutide (Gattex^®^)	GLP-2 receptor agonism	SC daily; approved (short bowel syndrome)	Regulatory precedent for regenerative GI peptide	Narrow approved indication; immunogenicity; high cost
BPC-157 (proposed)	BPC-157	Indirect cytoprotective/regenerative; Egr-1, NO-system, GH-receptor (target undefined)	Multi-route potential (oral, SC, IM, intra-articular, topical); investigational, no approval	Small-peptide manufacturing economics; route flexibility; regenerative without immunosuppression	Undefined molecular target; no validated human PK; no formulation; no controlled clinical data

**Table 4:** Formulation strategies applicable to BPC-157 across routes of administration, with mechanistic rationale and current development status. SNAC: sodium N-[8-(2-hydroxybenzoyl)amino]caprylate; SC: subcutaneous; IM: intramuscular; PLGA: poly(lactic-co-glycolic acid). Development status refers to published peer-reviewed evidence specific to BPC-157; strategies listed as “not investigated” have established evidence bases in other peptide therapeutics but have not been applied to BPC-157 in any published study.

Strategy	Route	Mechanism	Key Advantage	Development Status	Feasibility for BPC-157
Absorption enhancer (e.g., SNAC)	Oral	Local pH elevation; transcellular permeation	Clinically validated (semaglutide)	Not investigated for BPC-157	Highest
Chitosan nanoparticles	Oral	Mucoadhesion; tight junction opening	Dual permeation + protection	Not investigated	Moderate-to-high
PLGA microspheres	SC/IM depot	Sustained polymer erosion release	Weekly–monthly dosing	Not investigated	Lower (compatibility uncharacterised)
In situ forming gel	SC depot	Temperature/pH-triggered gelation	Simple manufacturing	Not investigated	Lower (compatibility uncharacterised
Hyaluronic acid hydrogel	Intra-articular	Extended synovial residence	Local delivery; approved excipient	Not investigated	High for local delivery
Enteric coating	Oral	Gastric bypass	Not indicated (gastric stable)	Not applicable	Not indicated

**Table 5:** Summary of published human studies of BPC-157. AEs: adverse event; T½: elimination half-life; IV: intravenous; PK: pharmacokinetics. Evidence levels assigned according to the Oxford Centre for Evidence-Based Medicine hierarchy. *n*: number of subjects enrolled.

Study	Year	*n*	Route	Indication	Design	Key Finding	Evidence Level
Lee & Burgess [10]	2025	2	IV	Safety/PK (healthy)	Uncontrolled pilot	No AEs; T½ <30 min	Level V
Vasireddi et al. [7]	2025	16	Intra-articular	Chronic knee pain	Retrospective	87.5% pain relief	Level IV
Lee et al. [52]	2024	12	Intravesical	Interstitial cystitis	Uncontrolled pilot	80–100% symptom resolution	Level IV

**Table 6:** Proposed translational development roadmap for BPC-157, organized by developmental phase with estimated timelines. LC-MS/MS: liquid chromatography–tandem mass spectrometry; ICH: International Council for Harmonisation; GMP: Good Manufacturing Practice; BCS: Biopharmaceutical Classification System; GLP: Good Laboratory Practice; IND: Investigational New Drug; SAD: single ascending dose; MAD: multiple ascending dose; ADME: absorption, distribution, metabolism, excretion; pop-PK: population pharmacokinetics; RCT: randomized controlled trial; NSAID: non-steroidal anti-inflammatory drug; IBD: inflammatory bowel disease. Estimated timelines are approximate and assume adequate sponsor resources and regulatory engagement; actual timelines may vary.

Phase	Activities	Key Deliverables	Estimated Timeline
Phase 0—Pharmaceutical foundation	LC-MS/MS bioanalytical method; ICH Q1A stress testing; solubility/permeability profiling; GMP drug substance manufacture	Validated bioanalytical method; drug substance specification; preliminary BCS classification	12–18 months
Phase 1—Nonclinical safety	GLP repeat-dose toxicity (rat, dog); ICH S7A/S7B safety pharmacology; genotoxicity battery; local tolerance	IND-ready nonclinical package	18–36 months
Phase 2—First-in-human PK	SAD/MAD study in healthy volunteers; full ADME; mass balance; pop-PK modeling	Human PK parameters; clinical dose justification	12–18 months post-Phase 1
Phase 3—Proof-of-concept	Phase IIa RCTs in 2–3 indications (e.g., NSAID enteropathy, Achilles tendinopathy, IBD)	Efficacy signal; dose–response; safety in patients	24–36 months post-Phase 2

**Table 7:** Biopharmaceutical Indication Prioritisation for BPC-157 Clinical Development: Route of Administration Feasibility and Development Priority. GI: gastrointestinal; IBD: inflammatory bowel disease; IM: intramuscular; IV: intravenous; CNS: central nervous system; PK: pharmacokinetics.

Indication	Biopharmaceutical Justification	Development Priority
NSAID enteropathy/IBD	Oral route; local GI effect; no systemic absorption required	First priority
Achilles tendinopathy	Local IM/intra-tendinous; circumvents PK limitations	First priority
Intra-articular knee pain	Local intra-articular; synovial compartment retention	First priority
Perioperative organ protection	IV route; single-dose; defined PK window	Second priority
Cardiovascular/vascular	Systemic; requires validated human PK first	Second priority
CNS indications	Systemic or intranasal; most biopharmaceutically complex	Third priority

## Data Availability

No new data were generated or analyzed in this study. All information is derived from previously published studies, which are cited within the manuscript.

## Abbreviations

The following abbreviations are used in this manuscript:

BPC-157	Body Protection Compound 157
ADME	Absorption, Distribution, Metabolism, and Excretion
BCS	Biopharmaceutical Classification System
PK	Pharmacokinetics
PD	Pharmacodynamics
PK/PD	Pharmacokinetic–Pharmacodynamic
VEGF	Vascular Endothelial Growth Factor
VEGFR2	Vascular Endothelial Growth Factor Receptor 2
Egr-1	Early Growth Response 1
NO	Nitric Oxide
eNOS	Endothelial Nitric Oxide Synthase
HO-1	Heme Oxygenase-1
TNF-α	Tumor Necrosis Factor Alpha
IL	Interleukin
NF-κB	Nuclear Factor Kappa B
LC-MS/MS	Liquid Chromatography–Tandem Mass Spectrometry
Cmax	Maximum Plasma Concentration
AUC	Area Under the Curve
IM	Intramuscular
IV	Intravenous
SC	Subcutaneous
CNS	Central Nervous System
PLGA	Poly(lactic-co-glycolic acid)
SNAC	Sodium N-[8-(2-hydroxybenzoyl)amino]caprylate
ICH	International Council for Harmonisation
FDA	Food and Drug Administration
EMA	European Medicines Agency
WADA	World Anti-Doping Agency
cGMP	Current Good Manufacturing Practice
GLP	Good Laboratory Practice
